# Opioid use after breast cancer in Denmark: a nationwide study of social and clinical factors

**DOI:** 10.1093/jnci/djag052

**Published:** 2026-02-25

**Authors:** Kirsten M Woolpert, Anders Kjærsgaard, Lidia Schapira, Henrik Toft Sørensen, Deirdre Cronin-Fenton

**Affiliations:** Department of Clinical Epidemiology, Department of Clinical Medicine, Aarhus University and Aarhus University Hospital, Aarhus, Denmark; Department of Clinical Epidemiology, Department of Clinical Medicine, Aarhus University and Aarhus University Hospital, Aarhus, Denmark; Department of Medicine, Division of Oncology, Stanford University School of Medicine, Palo Alto, CA, United States; Department of Clinical Epidemiology, Department of Clinical Medicine, Aarhus University and Aarhus University Hospital, Aarhus, Denmark; Department of Medicine, Division of Oncology, Stanford University School of Medicine, Palo Alto, CA, United States; Center for Population Medicine, Aarhus University and Aarhus University Hospital, Aarhus, Denmark; Clinical Excellence Research Center, Stanford University, Palo Alto, CA, United States; Department of Clinical Epidemiology, Department of Clinical Medicine, Aarhus University and Aarhus University Hospital, Aarhus, Denmark

## Abstract

**Background:**

Pain is a common, long-lasting effect of breast cancer treatment, but adequate pain management can be a challenge in survivorship care. Opioids remain important for acute and chronic pain, yet prolonged use carries risks of dependence and overdose. Denmark has historically had high opioid prescription rates, but the duration, strength, and intensity of use among breast cancer survivors is not well described.

**Methods:**

We conducted a nationwide registry-based study of 84 610 Danish women diagnosed with stage I to III breast cancer between 1997 and 2020. We described temporal trends in opioid prescribing and examined associations between sociodemographic and clinical characteristics and 5 potentially harmful opioid-related outcomes after breast cancer: new and prolonged use, long-term use, long-term strong use, concurrent use with sedative hypnotics, and diagnosed substance-related disorder or overdose.

**Results:**

Within 1 year of diagnosis, 18% of women filled at least 1 opioid prescription; this proportion declined steadily after 2015. Codeine, the most common initial opioid in the late 1990s, was gradually replaced by tramadol, morphine, and oxycodone. Across the cohort, 6.1% had long-term use, 2.0% had long-term strong use, 3.6% had concurrent opioid and sedative hypnotic use, and 0.4% developed a substance-related disorder or overdose. Among 74 771 opioid-naïve patients, 1.7% became new and prolonged users. The odds of these opioid-related behaviors were higher among women with socioeconomic hardships, psychiatric morbidity, comorbidity, and advanced disease.

**Conclusion:**

Opioid use after breast cancer was uncommon and declined over time in Denmark. Nonetheless, social and clinical disparities persisted, highlighting the importance of survivorship care that balances safe prescribing with adequate pain management.

## Introduction

Persistent pain after breast cancer treatment is common. Studies worldwide have reported that 20% to 60% of women experience pain long after completing therapy.^1^ In Denmark, nearly half (47%) of patients with breast cancer have pain that persists 2 to 3 years after surgery.^2^ This pain often has neuropathic characteristics because of surgical nerve injury, chemotherapy, or radiation effects^3^ and can substantially impair quality of life and daily function.^4^^,^^5^ For many survivors, pain persists well beyond cancer treatment and becomes a chronic consequence of cancer therapy.

Opioids remain important tools for managing moderate to severe chronic pain in breast cancer survivors.^6^ Nonetheless, prolonged use is a growing concern.^7^ Long-term or high-dose opioid therapy carries risks similar to those seen in populations with chronic noncancer pain, including dependence, overdose, and other adverse events.^8^ Risks are further heightened when opioids are used concurrently with sedative hypnotics, such as benzodiazepines, which can cause additive respiratory depression and increase overdose risk.^9^^,^^10^

Several countries, including Denmark, the United States, and Canada, have historically had high levels of opioid prescribing, although the outcomes and policy responses have differed.^11-13^ Denmark’s per-capita opioid consumption ranked among the top 5 globally by the late 2010s, with rates nearly twice those of neighboring Sweden and Norway.^14^^,^^15^ Liberal prescribing practices and widespread tramadol use contributed to this pattern.^16^^,^^17^ A series of national policies introduced between 2013 and 2019, including pack-size restrictions, reclassification of tramadol to a monitored controlled substance, and updated prescribing guidance, sought to decrease opioid use and address safety concerns.^17-19^

Even within this broader policy context, opioid prescribing and use are not evenly distributed across patients. In both cancer and noncancer settings, lower income, unemployment, and shorter educational attainment have been associated with higher rates of long-term opioid use, concurrent sedative hypnotic use, and opioid-related disorders.^20^^,^^21^ Clinical characteristics, including advanced disease stage at diagnosis, might also influence pain burden and opioid needs.^22^ In Denmark’s universal healthcare system, such disparities might reflect differences in underlying pain severity, prescribing practices, health literacy, or access to nonpharmacologic pain management.

To address these gaps, we aimed to characterize opioid use after breast cancer diagnosis in Denmark. Specifically, we aimed to (1) describe nationwide trends in prescribing opioids within the first 6 months after diagnosis, (2) estimate the prevalence of 5 opioid-related outcomes after breast cancer (new and prolonged use, long-term use, long-term strong use, concurrent use with sedative hypnotics, and diagnosed substance-related disorder or overdose), and (3) examine how sociodemographic and clinical factors were associated with these outcomes.

## Methods

### Data sources and study population

In Denmark, all residents are assigned a unique civil personal registration number at birth or immigration that enables accurate data linkage across nationwide health and administrative registries.^23^ We used these registries to establish a cohort of women diagnosed with primary breast cancer (*International Statistical Classification of Diseases, Tenth Revision [ICD-10]* code C50) between January 1, 1997, and December 31, 2020, through the Danish Cancer Registry.^24^ Reliable information about cancer treatment modalities (chemotherapy, radiation therapy, surgery, and hormone therapy), breast cancer recurrence, and metastatic disease during follow-up was not available in the linked registries used for this analysis.

Medication exposures were ascertained from the Danish National Prescription Registry, which contains information about all outpatient drug prescriptions dispensed at Danish pharmacies since 1995.^25^ We captured all filling of opioid prescriptions from 1996 onward, as defined by Anatomical Therapeutic Chemical codes starting with N02A or R05DA04. We restricted the cohort to individuals who survived, had no diagnosis with a second primary cancer within 1 year after diagnosis, and resided in Denmark for at least 1 year after diagnosis to allow for observation of early postdiagnostic and post-treatment opioid use.

**Figure 1.**
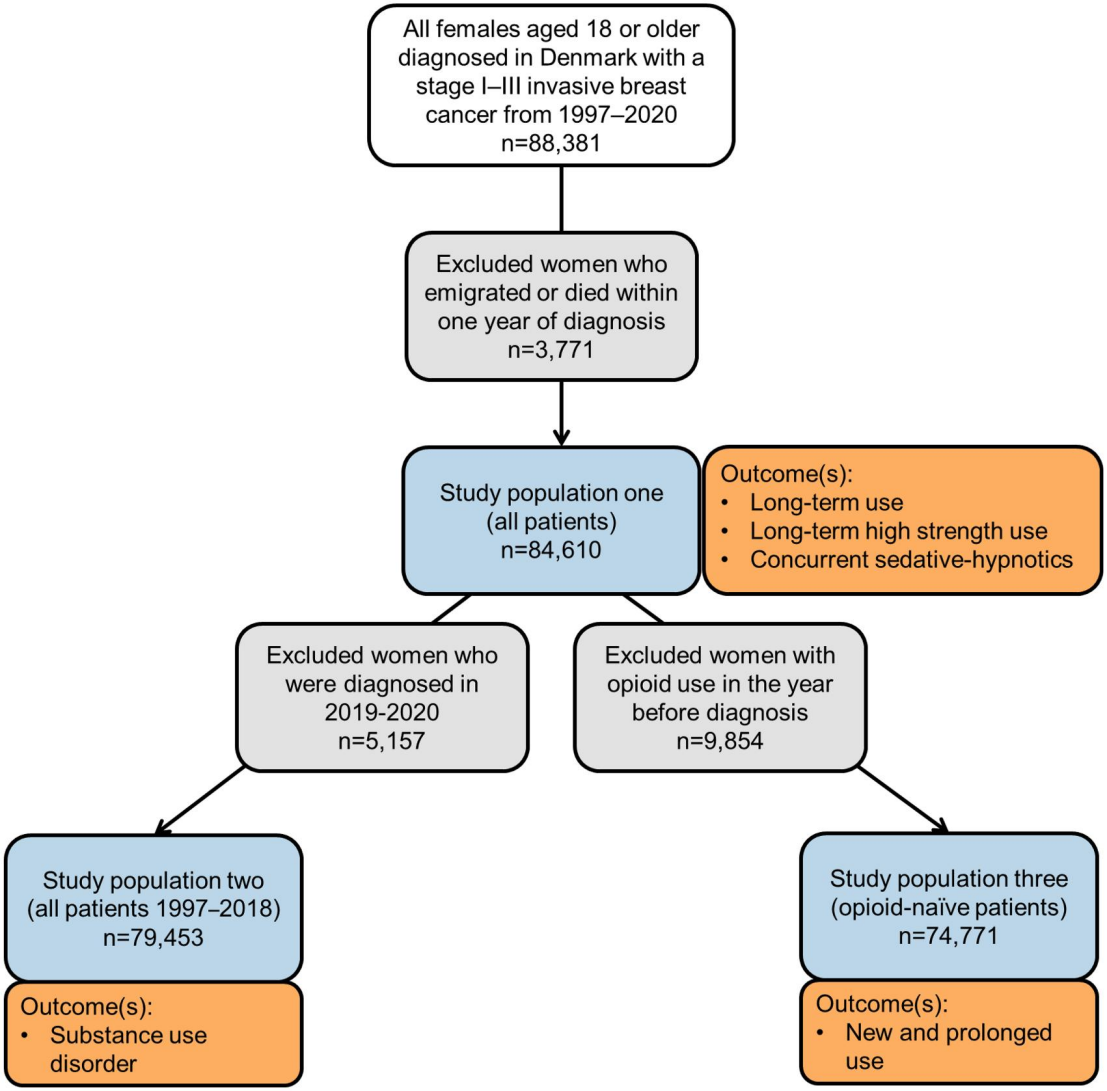
Flow chart for study inclusion for study population 1 (all patients), study population 2 (all patients, 1997-2018), and study population 3 (opioid-naïve patients).

From this base cohort, we defined 3 analytic study populations: (1) all eligible women diagnosed between 1997 and 2020 (study population 1), (2) the subset diagnosed between 1997 and 2018 to ensure complete follow-up (ie, 5 years, as data were available through 2023) for opioid-related disorders and overdose (study population 2), and (3) the subset with no opioid prescriptions in the year before breast cancer diagnosis (opioid-naïve patients; study population 3). A flowchart for inclusion and exclusion criteria for each population is provided in Figure 1.

### Exposures

We retrieved sociodemographic data at the time of diagnosis from the Danish Civil Registration System, including age at diagnosis, cohabitation status (cohabiting vs living alone), and marital status (married/registered partnership, not married/divorced, or widowed).^26^ Information about educational attainment (short, intermediate, long, or unknown) was obtained from the Danish Population’s Education Register.^27^ Information about employment status (working, not working, or retired) from 12 to 3 months before diagnosis was obtained from the Danish Registry for Evaluation of Marginalization.^28^ For each week during this period, employment status was registered, and we defined each woman’s overall status as the category occurring most frequently.^29^^,^^30^ Household income was obtained from the Danish Income Statistics Register^31^ and calculated as annual disposable income after tax, adjusted for household size and inflation, according to the median during the 2 years before breast cancer diagnosis. Quartiles were defined within 5-year age groups, and income was categorized as low (quartile 1), intermediate (quartiles 2-3), or high (quartile 4).^29^^,^^30^ If income data were missing for 1 of the 2 years, the available year was used; women with missing income in both years or with negative income in either year were excluded.^32^ Categorization and coding of social variables were defined as recommended by a recently published Danish national guideline.^33^

We ascertained data on cancer stage at diagnosis (I, II, or III) from the Danish Cancer Registry.^13^ Estrogen receptor status (positive, negative, or unknown/not measured) was obtained from the Danish Pathology Registry within 6 months after diagnosis for patients diagnosed from 2005 onward, when mandatory reporting of pathology results to the registry was introduced.^34^^,^^35^ In 2010, the estrogen receptor positivity level necessary to define estrogen receptor–positive disease was lowered from 10% to 1% and was accounted for in our definition.^36^ ERBB2 (formerly HER2) status was also captured in the 6 months after diagnosis but only for patients diagnosed from 2008, when routine ERBB2 testing became recommended by guidelines.^36^ The Charlson Comorbidity Index score was defined from all diagnoses in the Danish National Patient Registry in the 5 years before breast cancer diagnosis.^37^ Psychiatric diagnoses were defined in the same manner and also captured psychiatric hospitalizations from the Danish Psychiatric Central Register.^38^^,^^39^ Additional definitions and coding schemes are provided in Table S1.

### Outcomes

We defined 5 non–mutually exclusive opioid-related outcomes, adapted from the definitions described by Chao et al.,^40^ with modifications to fit the Danish registry setting. These outcomes were defined as follows:

Among the entire cohort (study population 1):

- *Long-term opioid use:* continuous opioid treatment lasting longer than 90 days within 1 year after diagnosis, allowing for a 30-day gap between fills
- *Long-term strong opioid use:* same as above but limited to prescriptions for opioids other than tramadol, codeine, or dextropropoxyphene^41^
- *Concurrent use of opioids and sedative hypnotics:* defined as overlapping prescriptions for opioids and benzodiazepines or other sedative hypnotic drugs (Anatomical Therapeutic Chemical codes starting with N05CD or N05CF) for 30 or more days within 1 year after breast cancer diagnosis

Among the entire cohort, restricted to women diagnosed between 1997 and 2018 (study population 2):

- *Substance-related disorder or overdose:* diagnoses of opioid-related disorder (*ICD-10* code F11), stimulant-related disorder (F15), psychoactive substance-related disorder (F19), or opioid poisoning (T40) were identified from the Danish National Patient Registry or the Danish Psychiatric Central Register within 5 years after breast cancer diagnosis; in addition, fatal overdoses were captured from the Danish Cause of Death Registry according to underlying cause-of-death codes (*ICD-10* codes X40–X44, X60–X64, X85, and Y10–Y14)

Among only the opioid-naïve cohort (study population 3):

- *New and prolonged use:* 1 or more opioid prescriptions within 6 months after diagnosis and 2 or more prescriptions during months 6 through 12 after diagnosis

### Statistical analysis

We first visually explored trends in opioid prescribing over the study period and described baseline cohort sociodemographic and clinical characteristics. These trends are reported from study population 1, which is the most representative of all breast cancer survivors in Denmark. We also reviewed Danish regulatory documents and published literature on opioid policy changes to qualitatively compare the timing of these initiatives with observed prescribing trends. Although our main outcome definitions do not account for opioid dose heterogeneity, we also examined the distribution of cumulative morphine milligram equivalents dispensed during the first year after diagnosis among opioid users.^42^^,^^43^ Finally, logistic regression was used to estimate odds ratios (ORs) and 95% CIs for the association between exposures and outcomes, with analyses conducted within the relevant study population. Because the aim of this study was descriptive rather than causal, we present estimates adjusted only for age at diagnosis and calendar year at diagnosis. Models were also stratified by calendar year at diagnosis.

Analyses were conducted in SAS, version 9.4, statistical software (SAS Institute Inc). Figures were generated in R, version 4.0, software (R Foundation for Statistical Computing). All data processing and analysis were performed within Statistics Denmark in accordance with the General Data Protection Regulation. This study was registered with Aarhus University 2022-0367531, No. 2979.

## Results

### Cohort characteristics

The main study population for descriptive analyses (study population 1) included 84 610 women diagnosed with stage I to III breast cancer in Denmark between 1997 and 2020 who survived and remained residents for at least 1 year after diagnosis (Table 1). Most (40%) were diagnosed with breast cancer between ages 50 and 64 years. At the time of diagnosis, 58% were married, and two-thirds were living with a partner. Analysis of socioeconomic characteristics indicated that 34% had short education durations, 35% had intermediate education durations, and 26% had long education durations. In the year before diagnosis, 64% of patients were working, 16% were not working, and 21% were retired.

**Table 1.** Baseline sociodemographic and clinical characteristics^a^ of 84 610 breast cancer survivors in Denmark, 1997-2020.

Characteristic^a^	Study population 1, all patients, No. (%)
Total cohort	84 610 (100)
**Sociodemographic characteristics**	
Age at diagnosis, y	
<40	3273 (3.9)
40-49	11 288 (13)
50-64	33 579 (40)
65-74	21 308 (25)
≥75	15 162 (18)
Marital status	
Married	49 336 (58)
Divorced	12 294 (15)
Unmarried	8195 (9.7)
Widowed	14 785 (17)
Cohabitation status	
Cohabiting	54 075 (64)
Living alone	30 535 (36)
Education status	
Short	28 349 (34)
Intermediate	29 903 (35)
Long	22 157 (26)
Unknown	4201 (5.0)
Employment status	
Working	53 929 (64)
Not working	13 163 (16)
Retired	17 518 (21)
Household income (quartile)	
Highest (>3)	20 993 (25)
Intermediate (2-3)	41 995 (50)
Lowest (<1)	20 990 (25)
Unknown^b^	632 (0.8)
**Clinical characteristics**	
Disease stage at diagnosis	
I	45 163 (53)
II	30 352 (36)
III	9095 (11)
Estrogen receptor status (after 2005)^c^	
Positive	39 430 (67)
Negative	12 521 (21)
Unknown/not measured	6720 (11)
ERBB2 status (after 2008)^d^	
Positive	5761 (12)
Negative	30 401 (63)
Unknown/not measured	12 244 (25)
Charlson Comorbidity Index	
None	71 195 (84)
1-2	11 918 (14)
≥3	1497 (1.8)
Psychiatric comorbidity	
None	81 560 (96)
Moderate	2144 (2.5)
Severe	906 (1.1)

a Characteristics were measured at or before the date of breast cancer diagnosis, except for estrogen receptor and ERBB2 status, which were captured within 6 months after breast cancer diagnosis.

b Women with missing or negative income data were categorized as unknown.

c Mandatory reporting of pathological examinations to the Danish Pathology Register began in 2005. Counts in these cells will not sum to the total cohort size.

d ERBB2 testing standardization began in 2008. Therefore, the numbers reported for ERBB2 testing are among the 41 903 patients diagnosed after the guideline change. Counts in these cells will not sum to the total cohort size.

In terms of clinical characteristics, 53% of patients had stage I disease, 36% had stage II disease, and 11% had stage III disease. Among women diagnosed after 2005, 67% had estrogen receptor–positive tumors, whereas 21% had estrogen receptor–negative tumors. Most patients had no recorded comorbidities: 84% had a Charlson Comorbidity Index score of 0. Psychiatric morbidity was uncommon: only 3.6% of patients had a moderate or severe psychiatric diagnosis. Characteristics among all 3 study cohorts were similar; characteristics among opioid-naïve patients (study population 3) are displayed in Table S2 and Figures S1 and S2. The distribution of cumulative morphine milligram equivalents in the first year after diagnosis among opioid users was skewed toward lower dose regimens (median = 800 mg [IQR = 300-2000 mg]) (Figure S3).

### Opioid prescribing patterns

**Figure 2.**
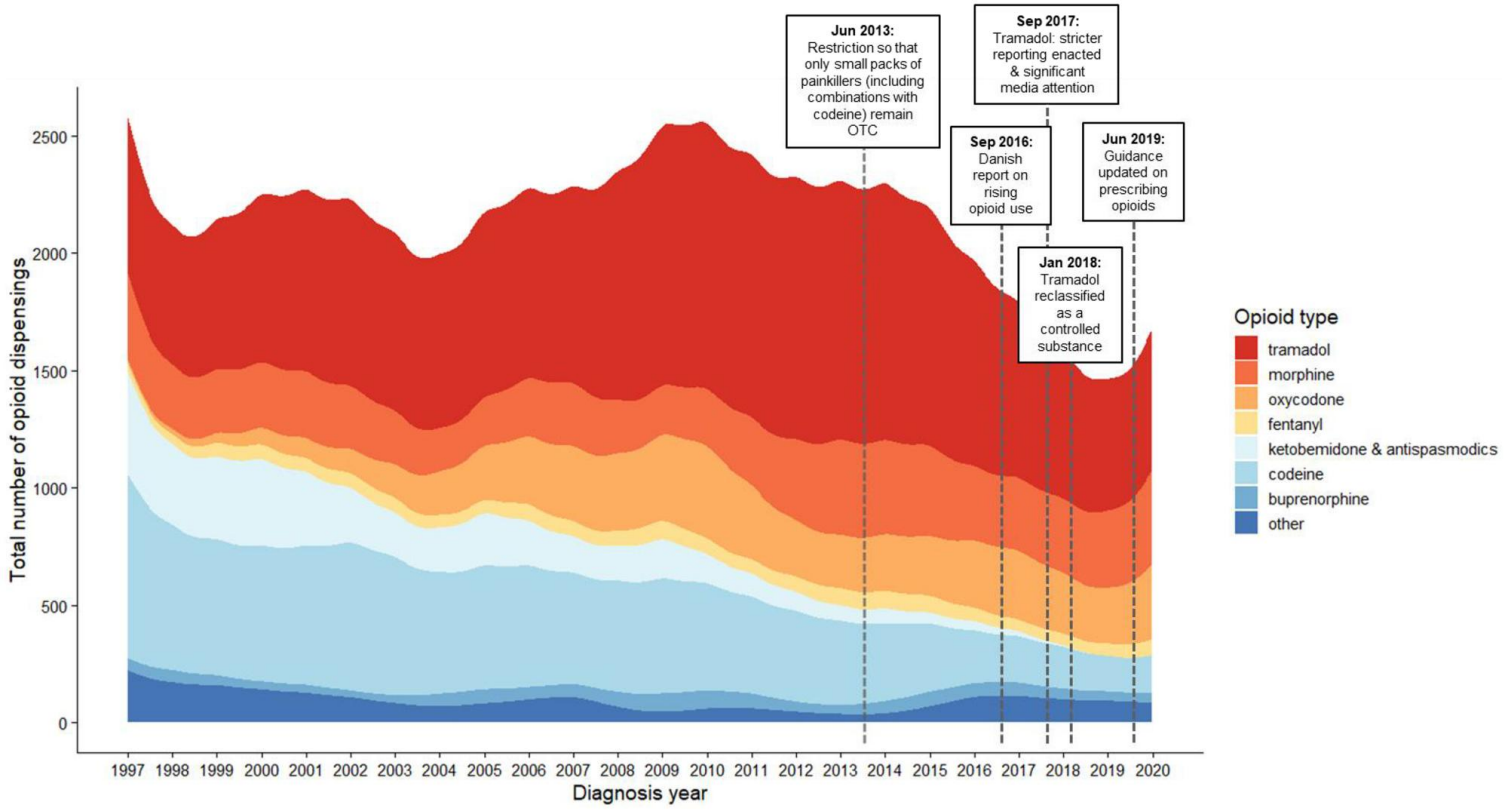
Trends in all opioid prescribing within 6 months of breast cancer diagnosis among 84 610 breast cancer survivors in Denmark, 1997-2020, shown in relation to national opioid policy and prescribing practice changes. Each stream represents the total number of opioid prescriptions filled within 6 months of diagnosis, stratified by drug type.

**Figure 3.**
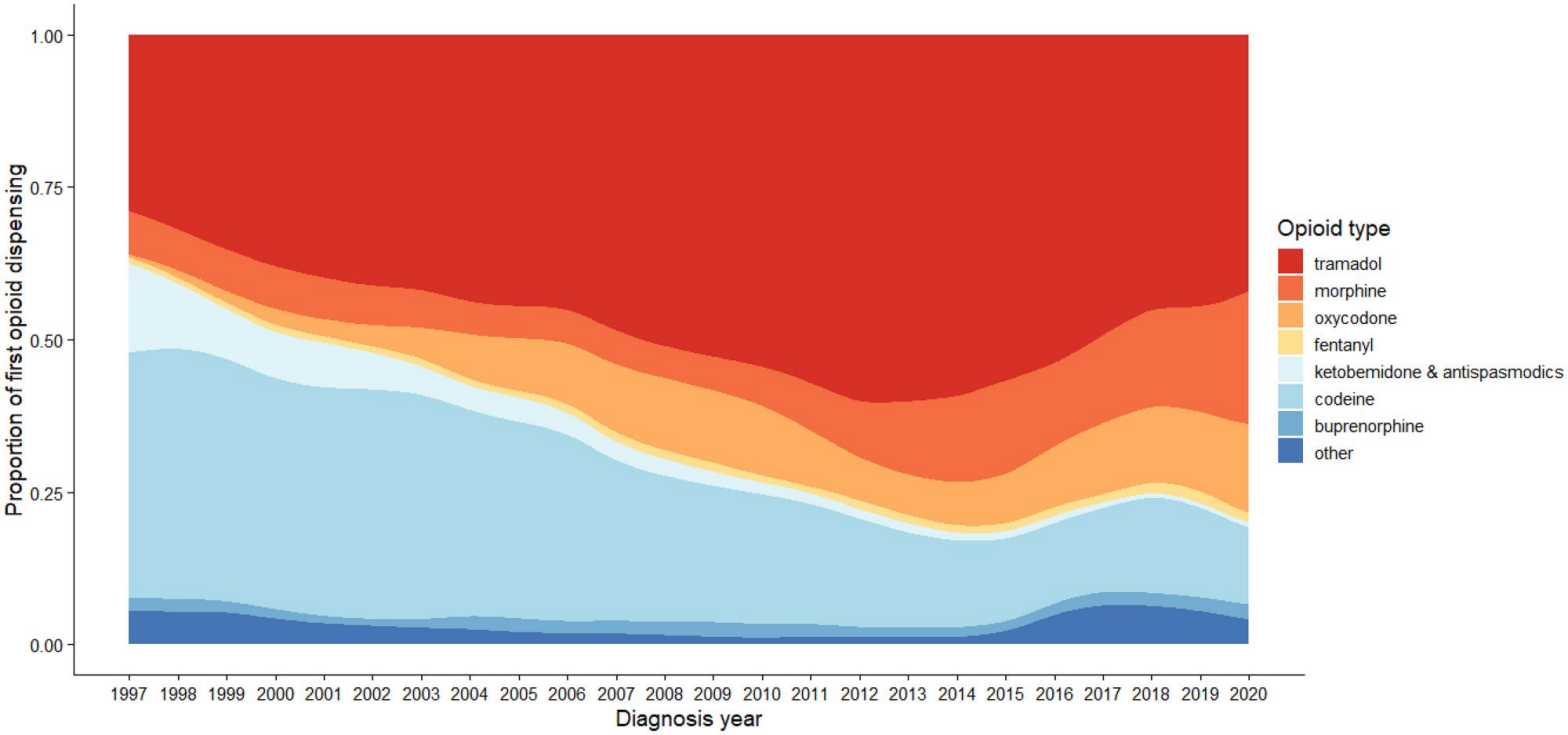
Trends in initial opioid prescription within 6 months of breast cancer diagnosis among 84 610 survivors in Denmark, 1997-2020. Each stream represents the proportion of survivors whose first opioid prescription within 6 months of diagnosis was for the specified drug. Proportions are based on only the first prescription and do not reflect subsequent changes in therapy.

Examination of all opioid prescriptions filled within 6 months after diagnosis indicated that overall dispensing has decreased over time, with a peak occurring around 2010 but ultimately reaching the lowest levels at the end of follow-up (Figure 2). Trends in the first type of opioid dispensed within 6 months after breast cancer diagnosis shifted over the study period (Figure 3). In the late 1990s, codeine was the most common first prescription. Its use began to decline in the early 2000s, coinciding with a steady rise in tramadol prescription. By the mid-2010s, morphine and oxycodone also accounted for a share of initial dispensing, whereas codeine use had become more uncommon.

### Opioid-related outcomes

**Figure 4.**
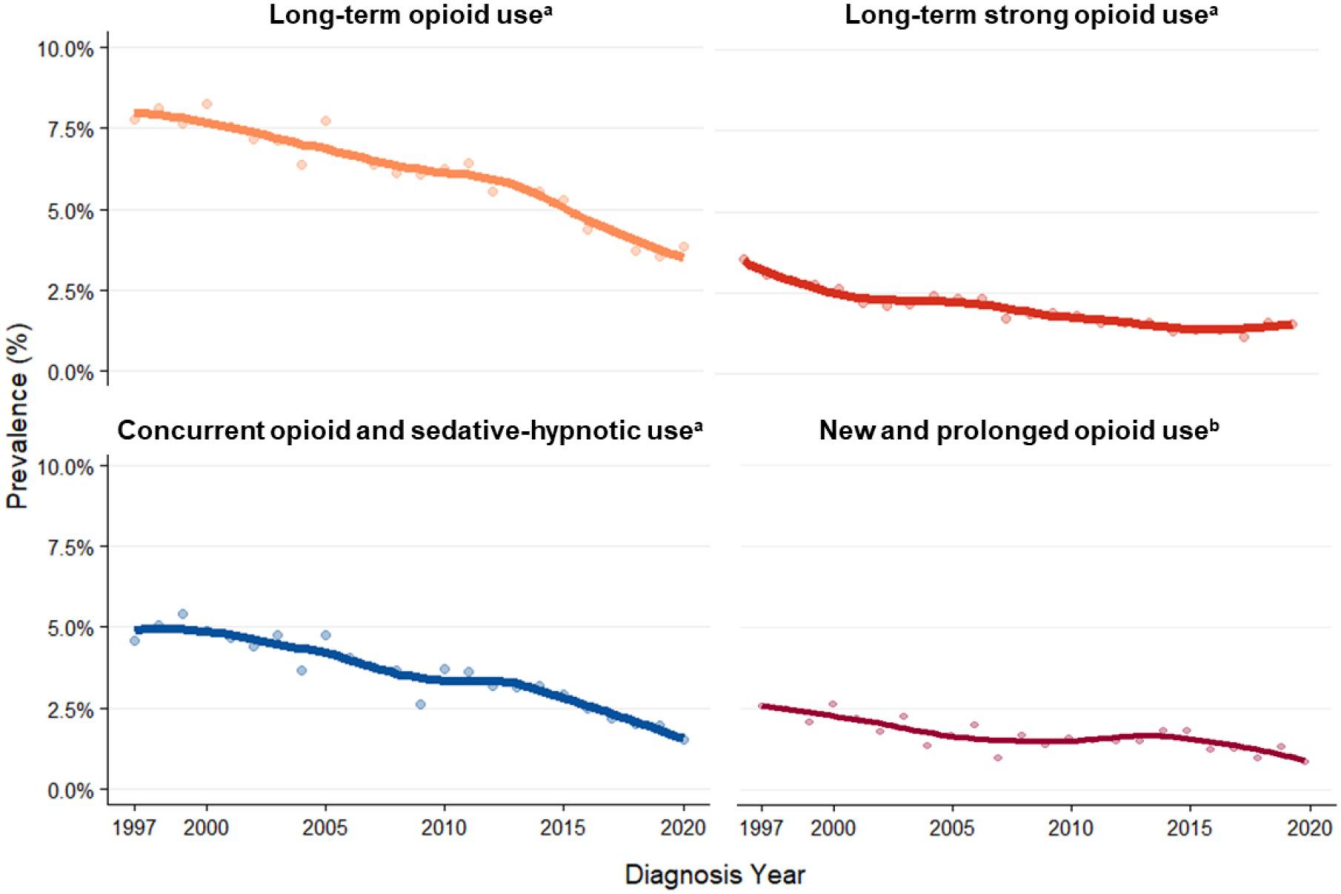
Trends in 4 opioid-related outcomes (not mutually exclusive) among breast cancer survivors in Denmark, 1997-2020. ^a^Long-term opioid use, long-term strong opioid use, and concurrent opioid and sedative hypnotic use were assessed among 84 610 survivors. ^b^New and prolonged opioid use was assessed among 74 771 opioid-naïve survivors.

Across the relevant study populations, 6.1% of all women had long-term opioid use, 2.0% had long-term strong opioid use, 3.6% had concurrent opioid and sedative hypnotic use, 0.4% were diagnosed with a substance-use disorder (SUD) or overdose, and 1.7% of opioid-naïve women became new and prolonged opioid users. The absolute numbers of these outcomes were low, and the prevalence of all outcomes declined steadily over time (Figure 4). Privacy regulations prevent presentation of exact figures for SUD or overdose, but prevalence remained low across the study period.

**Figure 5.**
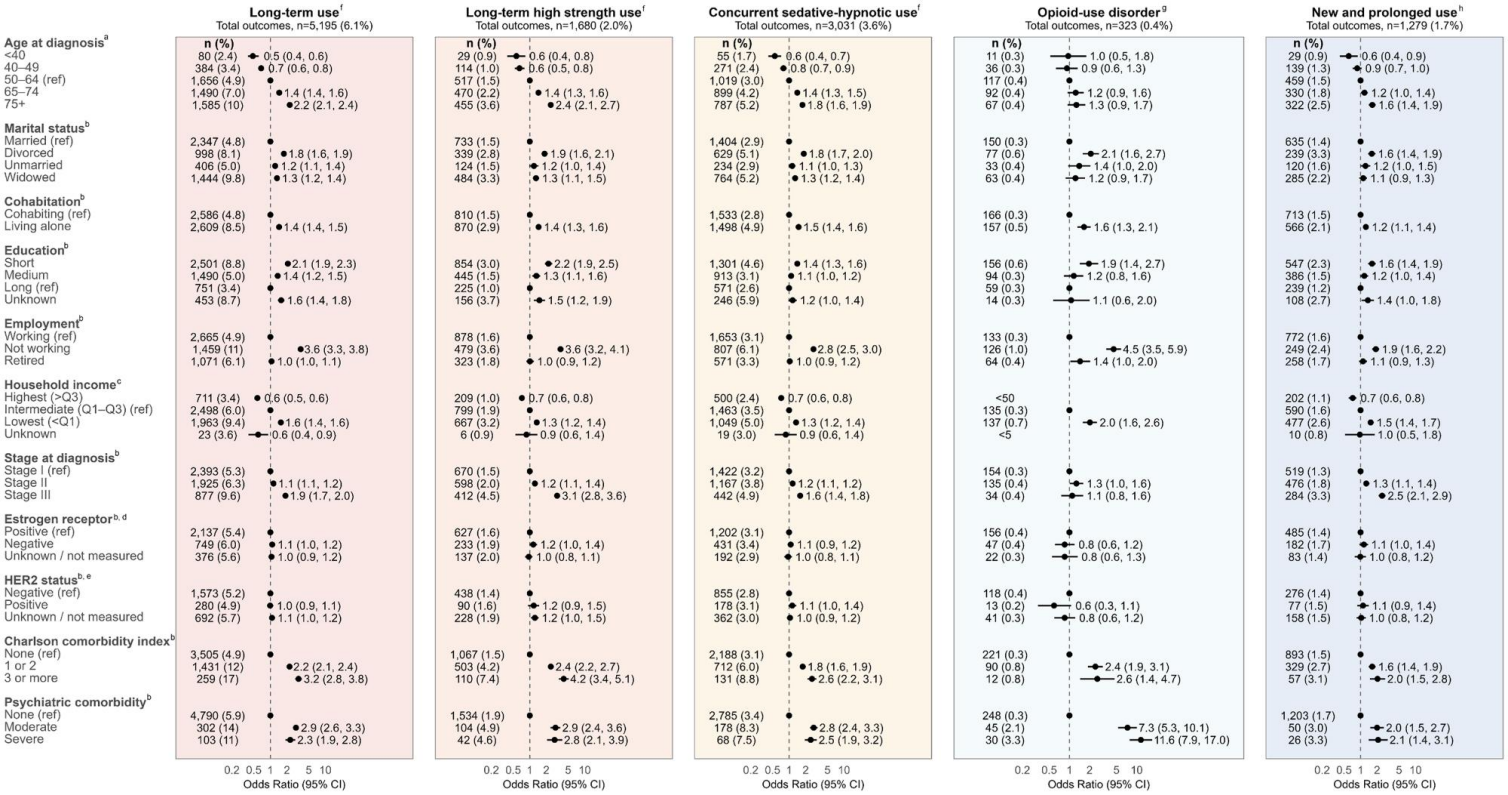
Associations of sociodemographic and clinical characteristics with 5 non–mutually exclusive opioid-related outcomes among breast cancer survivors in Denmark, 1997-2020. Q = quartile. ^a^Reported associations are unadjusted. ^b^Reported associations are adjusted for age at diagnosis and calendar year of diagnosis. ^c^Reported associations are adjusted only for calendar year of diagnosis because the definition for household income was standardized according to age at diagnosis. ^d^Mandatory reporting of pathological examinations to the Danish Pathology Register began in 2005. Outcome numbers will not sum to the reported total number of outcomes. ^e^ERBB2 testing standardization began in 2008. Outcome numbers will not sum to the reported total number of outcomes. ^f^Long-term use, long-term high-strength use, and concurrent sedative hypnotic use were assessed among 84 610 patients. ^g^Substance use disorder was assessed among the 79 453 patients diagnosed between 1997 and 2018 to ensure complete follow-up. ^h^New and prolonged use was assessed among 74 771 opioid-naïve patients. ^i^In accordance with Danish privacy laws, small cells and any cells that would allow for back-calculation have been masked.

Social and socioeconomic indicators were consistently associated with elevated risks of opioid-related outcomes (Figure 5). Compared with married women, divorced women had higher odds of new and prolonged opioid use (OR = 1.6, 95% CI = 1.4 to 1.9). Living alone was associated with elevated odds of long-term opioid use (OR = 1.4, 95% CI = 1.4 to 1.5), concurrent sedative hypnotic use (OR = 1.5, 95% CI = 1.4 to 1.6), and opioid use disorder (OR = 1.7, 95% CI = 1.3 to 2.1). Women with short education durations had higher risks of all outcomes, including opioid use disorder (OR = 2.0, 95% CI = 1.4 to 2.7) than women with long education durations. Being out of the job market at diagnosis was associated with long-term opioid use (OR = 3.6, 95% CI = 3.3 to 3.8) and SUD or overdose (OR = 4.6, 95% CI = 3.5 to 5.9). Women in the lowest income quartile also had elevated risks of long-term opioid use (OR = 1.6, 95% CI = 1.5 to 1.7) and SUD or overdose (OR = 2.0, 95% CI = 1.6 to 2.6).

Other patient and clinical characteristics were associated with increased risk of our opioid-related outcomes (Figure 5). Older women, particularly women 75 years of age or older, had higher odds of new and prolonged opioid use (OR = 1.6, 95% CI = 1.4 to 1.9) and long-term opioid use (OR = 2.3, 95% CI = 2.1 to 2.4) than women aged 50 to 64 years. Women with stage III disease had higher odds of new and prolonged opioid use (OR = 2.5, 95% CI = 2.1 to 2.9), long-term opioid use (OR = 1.9, 95% CI = 1.7 to 2.0), and long-term strong opioid use (OR = 3.2, 95% CI = 2.8 to 3.6) than patients with stage I disease. A greater comorbidity burden (Charlson Comorbidity Index score ≥3 compared with 0) was associated with higher risks, including new and prolonged opioid use (OR = 2.0, 95% CI = 1.5 to 2.8) and concurrent sedative hypnotic use (OR = 2.6, 95% CI = 2.2 to 3.1). Severe psychiatric illness was associated with new and prolonged opioid use (OR = 2.1, 95% CI = 1.4 to 3.1), long-term opioid use (OR = 2.3, 95% CI = 1.9 to 2.8), and SUD or overdose (OR = 11.6, 95% CI = 7.9 to 17.0). There were no meaningful differences in associations when stratified by calendar period (Tables S3A-S3C).

## Discussion

In this nationwide cohort of more than 84 000 Danish breast cancer survivors, opioid prescribing within 6 months after diagnosis became less common during more than 2 decades of follow-up. Codeine, once the dominant first prescription, was gradually replaced by tramadol, morphine, and oxycodone, but overall opioid dispensing declined after 2015. The prevalence of new and prolonged opioid use, long-term opioid use, strong opioid use, concurrent sedative hypnotic use, and diagnosed opioid-related disorders or overdoses all decreased during the study period, and the absolute risks were consistently low. These findings suggest that although opioids remain part of pain management in breast cancer survivorship, the occurrence of sustained opioid use has declined over time.

The decline in prescribing and opioid-related outcomes coincided with major policy initiatives in Denmark between 2013 and 2019, including restrictions on mild analgesic pack sizes, reclassification of tramadol, and updated national prescribing guidance.^14^^,^^18^^,^^19^ Growing awareness of opioid harms among clinicians and the public was likely to have reinforced these changes, thereby contributing to a more cautious approach to pain management.^17^^,^^44^^,^^45^ From an international perspective, Denmark’s experience illustrates how system-level regulation can shape opioid use. In the United States and Canada, liberal long-term prescribing contributed to a public health crisis of dependence, overdose, and mortality.^46^ In contrast, although prescribing rates have been high in European countries such as Denmark and Sweden, opioid-related deaths have not reached the same scale as observed in the United States (nearly 10 times fewer deaths than observed in the United States in 2017).^46^^,^^47^ This finding is likely to reflect Denmark’s universal healthcare system and restrictions on specific drugs. Nonetheless, high opioid consumption remains a concern in Europe, and socioeconomic differences in opioid use have also been documented in other settings.^46^^,^^47^

Our findings add to the evidence base by indicating that even in a regulated environment, vulnerable groups of breast cancer survivors face elevated risk of high-risk opioid use. Although the overall prevalence of the investigated opioid-related outcomes was low, we identified consistent disparities according to socioeconomic circumstances. Women with lower education or lower incomes or women outside the job market had a higher likelihood of becoming prolonged or long-term opioid users and developing opioid-related disorders. Living alone or being divorced or widowed was also associated with elevated risk. These findings have also been reported in other settings^48^^,^^49^ and suggest that survivorship care is shaped by patients’ broader life circumstances. Factors such as social support and economic security may influence both the experience of pain and the resources available to manage it.^50^^,^^51^

Clinical factors were used to further stratify the odds of potentially harmful opioid use. Women with advanced-stage disease or greater comorbidity were more likely to rely on opioids for prolonged periods, likely reflecting a greater pain burden. Older women also had elevated risks of sustained use, and women with psychiatric illness were particularly vulnerable. Mental health disorders are well-established risk factors for chronic opioid use and substantially increase the likelihood of opioid misuse. For example, depression and other psychiatric illnesses are associated with elevated odds of developing opioid dependence and overdose.^52^^,^^53^ In a large survey, nearly two-thirds of adults with opioid use disorder had a co-occurring mental illness, and more than one-quarter were classified as having serious mental illness.^54^ This population might be particularly susceptible to not only persistent pain but also misuse while also facing barriers to nonpharmacologic treatment options.^55^

Together, these results suggest that national policies can decrease overall prescribing, but patient-level vulnerabilities continue to shape who remains at risk. Denmark’s experience suggests that regulation alone cannot eliminate inequities and that survivorship care must address both clinical and social circumstances. For international audiences, this study provides evidence that high opioid prescribing does not inevitably correlate with widespread harm. It also emphasizes that decreased prescribing must be balanced against the risk of undertreatment, particularly among vulnerable groups. Integrating safe prescribing with equitable access to pain management will be essential to ensure recovery for all breast cancer survivors, regardless of social or clinical background.

This study has limitations. Because the prescription fill data captured dispensing at community pharmacies rather than actual consumption, some women may not have taken the medications as prescribed. Moreover, data on inpatient opioid use were not available. A major limitation is the lack of detailed clinical information about cancer treatment and disease course. Specifically, we lacked information about treatment modalities that may influence pain as well as information about breast cancer recurrence or metastatic disease during follow-up. As a result, we could not evaluate treatment-related pain, pain severity, or chronicity; distinguish cancer-related from non–cancer-related indications for opioid use; or assess whether opioid prescribing patterns differed by disease progression or treatment intensity, which limits the clinical interpretation of our findings. Opioid-related disorders are also likely to be underdiagnosed, particularly among women with stigma-related barriers to care.^56^^,^^57^ In addition, we did not aim for extensive covariate adjustment because our goal was to provide a descriptive account of opioid prescribing rather than to estimate causal effects. Finally, for outcomes assessed over 5 years, death may represent a competing risk, particularly among older women, and our logistic modeling approach did not account for differential time to death, which may have influenced the observed associations.

In conclusion, overall opioid use after breast cancer diagnosis has been uncommon and has declined over time in Denmark, but social and clinical disparities remain. System-level regulation may mitigate overall risks, yet tailored survivorship care may be needed to support women with socioeconomic hardships, psychiatric morbidity, and advanced disease and women who lack social support.

## Supplementary Material

djag052_Supplementary_Data

## Data Availability

The data that support the findings of this study are available from Danish registries, but restrictions apply to the availability of these data, which were used under license for the present study and so are not publicly available. Data are, however, available from the authors upon reasonable request and with permission.

## References

[1] Strijbos BTM , Janssen L, Voogd AC, Zwaans WAR, Roumen RMH, Maaskant-Braat AJG. Persistent pain after breast cancer treatment, an underreported burden for breast cancer survivors. Ann Surg Oncol. 2024;31:6753-6763. 38940899 10.1245/s10434-024-15682-2 PMC11413048

[2] Gärtner R , Jensen M-B, Nielsen J, Ewertz M, Kroman N, Kehlet H. Prevalence of and factors associated with persistent pain following breast cancer surgery. JAMA. 2009;302:1985-1992. 19903919 10.1001/jama.2009.1568

[3] Zachariae R , Christiansen P, Amidi A, et al The time has come for national clinical practice guidelines for managing late effects after cancer and cancer treatment. Acta Oncol. 2024;63:491-493. 38910334 10.2340/1651-226X.2024.40787 PMC11332514

[4] Caffo O , Amichetti M, Ferro A, Lucenti A, Valduga F, Galligioni E. Pain and quality of life after surgery for breast cancer. Breast Cancer Res Treat. 2003;80:39-48. 12889597 10.1023/A:1024435101619

[5] Hamood R , Hamood H, Merhasin I, Keinan-Boker L. Chronic pain and other symptoms among breast cancer survivors: prevalence, predictors, and effects on quality of life. Breast Cancer Res Treat. 2018;167:157-169. 28861642 10.1007/s10549-017-4485-0

[6] Swarm RA , Abernethy AP, Anghelescu DL, et al; National Comprehensive Cancer Network. Adult cancer pain. J Natl Compr Canc Netw. 2013;11:992-1022. 23946177 10.6004/jnccn.2013.0119 PMC5915297

[7] Shen C , Thornton JD, Gu D, et al Prolonged opioid use after surgery for early‐stage breast cancer. Oncologist. 2020;25:e1574–82-e1582. 32390251 10.1634/theoncologist.2019-0868 PMC7543235

[8] Winn AN , Check DK, Farkas A, Fergestrom NM, Neuner JM, Roberts AW. Association of current opioid use with serious adverse events among older adult survivors of breast cancer. JAMA Netw Open. 2020;3:e2016858. 32930779 10.1001/jamanetworkopen.2020.16858 PMC7492912

[9] Jann M , Kennedy WK, Lopez G. Benzodiazepines: a major component in unintentional prescription drug overdoses with opioid analgesics. J Pharm Pract. 2014;27:5-16. 24436437 10.1177/0897190013515001

[10] Bachhuber MA , Hennessy S, Cunningham CO, Starrels JL. Increasing benzodiazepine prescriptions and overdose mortality in the United States, 1996–2013. Am J Public Health. 2016;106:686-688. 26890165 10.2105/AJPH.2016.303061 PMC4816010

[11] Jayawardana S , Forman R, Johnston-Webber C, et al Global consumption of prescription opioid analgesics between 2009-2019: a country-level observational study. eClinicalMedicine. 2021;42:101198. 34820610 10.1016/j.eclinm.2021.101198 PMC8599097

[12] Cherny NI , Cleary J, Scholten W, Radbruch L, Torode J. The Global Opioid Policy Initiative (GOPI) project to evaluate the availability and accessibility of opioids for the management of cancer pain in Africa, Asia, Latin America and the Caribbean, and the Middle East: introduction and methodology. Ann Oncol 2013;24(suppl 11):xi7-xi13. 24436961 10.1093/annonc/mdt498

[13] Degenhardt L , Charlson F, Mathers B, et al The global epidemiology and burden of opioid dependence: results from the Global Burden of Disease 2010 Study. Addiction. 2014;109:1320-1333. 24661272 10.1111/add.12551

[14] Nissen SK , Pottegård A, Ryg J. Trends of opioid utilisation in Denmark: a nationwide study. Drugs Real World Outcomes. 2019;6:155-164. 31535353 10.1007/s40801-019-00163-w PMC6879688

[15] Groth Clausen T , Eriksen J, Borgbjerg FM. Legal opioid consumption in Denmark 1981-1993. Eur J Clin Pharmacol. 1995;48:321-325. 8641317 10.1007/BF00194945

[16] Rasmussen L , Ernst MT, Forberg S, Pottegård A, Søndergaard J, Sørensen AMS. Trends in utilization of tramadol and other opioids in Denmark 2017-2023: a nationwide drug utilization study. Br J Clin Pharmacol. 2025;91:908-913. 39825515 10.1111/bcp.16360

[17] Sørensen AMS , Rasmussen L, Ernst MT, et al Use of tramadol and other analgesics following media attention and risk minimization actions from regulators: a Danish nationwide drug utilization study. Eur J Clin Pharmacol. 2021;77:617-624. 33112987 10.1007/s00228-020-03016-6 PMC7935826

[18] Danish Medicines Agency (Lægemiddelstyrelsen). Painkillers to be prescription only in Denmark. 2013. Accessed September 18, 2025. https://laegemiddelstyrelsen.dk/en/news/reassessment-of-reimbursement-of-medicines-news-archives/painkillers-to-be-prescription-only-in-denmark/

[19] Danish Medicines Agency (Lægemiddelstyrelsen). Addictive drugs–revised guidance, frequently asked questions and new citizen leaflet. 2019. Accessed September 18, 2025. https://www.sst.dk/da/udgivelser/2019/Rationel-Farmakoterapi-5-2019/

[20] Do VM , Simpson S, Fisch KM, Gabriel RA. Associations between social determinants of health and opioid-use disorder among chronic pain patients from a multi-institutional dataset. Anesth Analg. 2025;141:1149-1158. 39715077 10.1213/ANE.0000000000007247 PMC12183315

[21] Marwitz KK , Noureldin M. A descriptive analysis of concomitant opioid and benzodiazepine medication use and associated adverse drug events in United States adults between 2009 and 2018. Explor Res Clin Soc Pharm. 2022;5:100130. 35478505 10.1016/j.rcsop.2022.100130 PMC9031034

[22] Gallaway MS , Townsend JS, Shelby D, Puckett MC. Pain among cancer survivors. Prev Chronic Dis. 2020;17:E54. 32644924 10.5888/pcd17.190367 PMC7367076

[23] Frank L. Epidemiology. When an entire country is a cohort. Science. 2000;287:2398-2399. 10766613 10.1126/science.287.5462.2398

[24] Gjerstorff ML. The Danish cancer registry. Scand J Public Health. 2011;39:42-45. 21775350 10.1177/1403494810393562

[25] Pottegård A , Schmidt SAJ, Wallach-Kildemoes H, Sørensen HT, Hallas J, Schmidt M. Data resource profile: the Danish national prescription registry. Int J Epidemiol. 2017;46:798-798f. 27789670 10.1093/ije/dyw213 PMC5837522

[26] Schmidt M , Pedersen L, Sørensen HT. The Danish civil registration system as a tool in epidemiology. Eur J Epidemiol. 2014;29:541-549. 24965263 10.1007/s10654-014-9930-3

[27] Jensen VM , Rasmussen AW. Danish education registers. Scand J Public Health. 2011;39:91-94. 21775362 10.1177/1403494810394715

[28] Hjollund NH , Larsen FB, Andersen JH. Register-based follow-up of social benefits and other transfer payments: accuracy and degree of completeness in a Danish interdepartmental administrative database compared with a population-based survey. Scand J Public Health. 2007;35:497-502. 17852980 10.1080/14034940701271882

[29] Hjorth CF , Damkier P, Ejlertsen B, Lash T, Sørensen HT, Cronin-Fenton D. Socioeconomic position and prognosis in premenopausal breast cancer: a population-based cohort study in Denmark. BMC Med. 2021;19:235. 34587961 10.1186/s12916-021-02108-z PMC8482675

[30] Schmidt JA , Woolpert KM, Hjorth CF, Farkas DK, Ejlertsen B, Cronin-Fenton D. Social characteristics and adherence to adjuvant endocrine therapy in premenopausal women with breast cancer. J Clin Oncol. 2024;42:3300-3307. 38917383 10.1200/JCO.23.02643

[31] Baadsgaard M , Quitzau J. Danish registers on personal income and transfer payments. Scand J Public Health. 2011;39:103-105. 21775365 10.1177/1403494811405098

[32] Ejlskov L , Plana-Ripoll O. Income in epidemiological research: a guide to measurement and analytical treatment with a case study on mental disorders and mortality. J Epidemiol Community Health. 2025;79:560-568. 39947873 10.1136/jech-2024-223206

[33] Hjorth CF , Kjærulff TM, Thomsen MK, et al; SEPLINE Group. SEPLINE: socioeconomic position in epidemiological research-a national guideline on Danish registry data. Clin Epidemiol. 2025;17:593-624. 40641826 10.2147/CLEP.S520772 PMC12242267

[34] Bjerregaard B , Larsen OB. The Danish pathology register. Scand J Public Health. 2011;39:72-74. 21775357 10.1177/1403494810393563

[35] Danish Health Data Authority (Sundhedsdatastyrelsen). Registrering af patologiske prøver. 2020. Accessed September 4, 2025. https://sundhedsdatastyrelsen.dk/indberetning/patientregistrering/registrering-specifikke-omraader/patologiske-proever

[36] Jensen MB , Laenkholm AV, Offersen BV, et al The clinical database and implementation of treatment guidelines by the Danish Breast Cancer Cooperative Group in 2007–2016. Acta Oncologica. 2018;57:13-18. 29202621 10.1080/0284186X.2017.1404638

[37] Charlson ME , Pompei P, Ales KL, MacKenzie CR. A new method of classifying prognostic comorbidity in longitudinal studies: development and validation. J Chronic Dis. 1987;40:373-383. 3558716 10.1016/0021-9681(87)90171-8

[38] Mors O , Perto GP, Mortensen PB. The Danish psychiatric central research register. Scand J Public Health. 2011;39:54-57. 21775352 10.1177/1403494810395825

[39] Møller J-JK , la Cour K, Pilegaard MS, Möller S, Jarlbaek L. Identification of socially vulnerable cancer patients - development of a register-based index (rSVI). Support Care Cancer. 2022;30:5277-5287. 35275294 10.1007/s00520-022-06937-3

[40] Chao CR , Zhou H, Shalman DM, et al Chronic opioid use and incident opioid use disorders in survivors of adolescent and young adult cancer after cancer treatment. Cancer. 2025;131:e35866. 40359220 10.1002/cncr.35866

[41] Cronin-Fenton DP , Heide-Jørgensen U, Ahern TP, et al Opioids and breast cancer recurrence: a Danish population-based cohort study. Cancer. 2015;121:3507-3514. 26207518 10.1002/cncr.29532 PMC4575607

[42] Rolová G , Skurtveit S, Handal M, et al Trends in opioid prescribing in Scandinavian countries from 2010 to 2023: insights from multi-metric evaluation. Br J Clin Pharmacol. 2025;91:3341-3352. 40702924 10.1002/bcp.70177 PMC12648362

[43] Stisen MG , Pedersen AB, Sheehan KJ, Mechlenburg I. Trajectories of opioid use following revision total hip arthroplasty: a population-based cohort study. J Arthroplasty. 2025;40:3246-3253.e11. 40436075 10.1016/j.arth.2025.05.081

[44] Nansen L , Viborg N. Morfinpillens skyggeside [Documentary]. DR2; 2017. Accessed September 18, 2025. https:// www.dr.dk/tv/se/afhaengighed-morfin-1-dr2dokumentar-2017/

[45] Nansen L , Viborg N. Smerter til salg - min kamp mod pillerne [Documentary]. DR2; 2017. Accessed September 18, 2025. https://www.dr.dk/tv/se/afhaengighedorfin-dr2dokumentar-morfinpillens-kyggeside/smerter-il-alginampod-llerne

[46] Ayoo K , Mikhaeil J, Huang A, Wąsowicz M. The opioid crisis in North America: facts and future lessons for Europe. Anaesthesiol Intensive Ther. 2020;52:139-147. 32419434 10.5114/ait.2020.94756 PMC10176520

[47] European Monitoring Centre for Drugs and Drug Addiction (EMCDDA). European Drug Report: Trends and Development. Publications Office of the European Union, Luxembourg. 2017. Accessed September 18, 2025. https://www.drugsandalcohol.ie/27401/1/European_Drug_Report_2017_FINAL.pdf

[48] Altekruse SF , Cosgrove CM, Altekruse WC, Jenkins RA, Blanco C. Socioeconomic risk factors for fatal opioid overdoses in the United States: findings from the Mortality Disparities in American Communities study (MDAC). PLoS One. 2020;15:e0227966. 31951640 10.1371/journal.pone.0227966 PMC6968850

[49] Lanier WA , Johnson EM, Rolfs RT, Friedrichs MD, Grey TC. Risk factors for prescription opioid-related death, Utah, 2008-2009. Pain Med. 2012;13:1580-1589. 23137228 10.1111/j.1526-4637.2012.01518.x

[50] Weiß M , Jachnik A, Lampe EC, et al Differential effects of everyday-life social support on chronic pain. BMC Neurol. 2024;24:301. 39198777 10.1186/s12883-024-03792-z PMC11351827

[51] Kapos FP , Craig KD, Anderson SR, et al Social determinants and consequences of pain: toward multilevel, intersectional, and life course perspectives. J Pain. 2024;25:104608. 38897311 10.1016/j.jpain.2024.104608 PMC11402600

[52] Sullivan MD , Edlund MJ, Steffick D, Unützer J. Regular use of prescribed opioids: association with common psychiatric disorders. Pain. 2005;119:95-103. 16298066 10.1016/j.pain.2005.09.020

[53] van Draanen J , Tsang C, Mitra S, et al Mental disorder and opioid overdose: a systematic review. Soc Psychiatry Psychiatr Epidemiol. 2022;57:647-671. 34796369 10.1007/s00127-021-02199-2 PMC8601097

[54] Jones CM , McCance-Katz EF. Co-occurring substance use and mental disorders among adults with opioid use disorder. Drug Alcohol Depend. 2019;197:78-82. 30784952 10.1016/j.drugalcdep.2018.12.030

[55] Onwumere J , Stubbs B, Stirling M, et al Pain management in people with severe mental illness: an agenda for progress. Pain. 2022;163:1653-1660. 35297819 10.1097/j.pain.0000000000002633 PMC9393797

[56] Goodyear K , Haass-Koffler CL, Chavanne D. Opioid use and stigma: the role of gender, language and precipitating events. Drug Alcohol Depend. 2018;185:339-346. 29499554 10.1016/j.drugalcdep.2017.12.037 PMC6097242

[57] Chou JL , Patton R, Cooper-Sadlo S, et al Stigma and Medication for Opioid Use Disorder (MOUD) among women. Int J Ment Health Addict. 2022;20:3262-3273.

