# Supplementary material for: Opioid use after breast cancer in Denmark: a nationwide study of social and clinical factors
Source: J Natl Cancer Inst. 2026 Feb 25;118(7):1257–65. doi: 10.1093/jnci/djag052 (PMC13339079; doi:10.1093/jnci/djag052)
Supplement: djag052_Supplementary_Data [file djag052_supplementary_data.docx]

**Supplementary Materials**

Opioid use after breast cancer diagnosis in Denmark: A nationwide study of social and clinical factors

**Authors:** Kirsten M Woolpert (1), Anders Kjærsgaard (1), Lidia Schapira (2), Henrik Toft Sørensen (1,2), Deirdre Cronin-Fenton (1)

Contents

[Table S1. Exposure definitions and data sources in Statistics Denmark 2](#_Toc219375790)

[Table S2. Baseline sociodemographic and clinical characteristics^a^ of 74,711 opioid-naïve breast cancer survivors (study population three) in Denmark, 1997–2020. 4](#_Toc219375791)

[Figure S1. Trends in all opioid prescribing within 6 months of breast cancer diagnosis among 74,711 opioid-naïve survivors (study population two) in Denmark, 1997–2020, shown in relation to national opioid policy and prescribing practice changes. 5](#_Toc219375792)

[Figure S2. Trends in initial opioid prescription within 6 months of breast cancer diagnosis among 74,711 opioid-naïve survivors (study population two) in Denmark, 1997–2020. 6](#_Toc219375793)

[Figure S3. Distribution of cumulative morphine milligram equivalents (MME) among 13,896 breast cancer survivors with at least one opioid dispensing in the year after diagnosis 7](#_Toc219375794)

[Table S3A. Associations of sociodemographic and clinical characteristics with four non-mutually exclusive opioid-related outcomes among 27,405 breast cancer survivors in Denmark, 1997–2004 8](#_Toc219375795)

[Table S3B. Associations of sociodemographic and clinical characteristics with four non-mutually exclusive opioid-related outcomes among 31,266 breast cancer survivors in Denmark, 2005–2012 9](#_Toc219375796)

[Table S3C. Associations of sociodemographic and clinical characteristics with four non-mutually exclusive opioid-related outcomes among 27,405 breast cancer survivors in Denmark, 2013–2020 10](#_Toc219375797)

[References 11](#_Toc219375798)

# Table S1. Exposure definitions and data sources in Statistics Denmark

| Exposure | Database | Variable | Codes |
| --- | --- | --- | --- |
| Marital status | Danish Civil Registration System | CIVST | Categorization adapted from the Danish SEPLINE national guideline:^1^   - Married or registered partnership: A, G, P - Widowed: E, L - Divorced: F, O - Unmarried: U |
| Cohabitation status | Danish Civil Registration System | FAMILIE_TYPE | Categorization adapted from the Danish SEPLINE national guideline:^1^   - Cohabiting: 1, 7, 2, 3, 4, 8 - Living alone: 5, 9, 10 |
| Education | Danish Population’s Education Registry | HFAUDD (formatted to ISCED by Statistics Denmark) | Categorization adapted from the Danish SEPLINE national guideline:^1^   - Short education: ISCED levels 1 and 2 - Medium education: ISCED levels 3 and 4 - Long education: ISCED levels 5, 6, 7 and 8 |
| Employment status | Danish Registry for Evaluation of Marginalization (DREAM) | y_YYWW | Categorization adapted from the Danish SEPLINE national guideline:^1^   - Working: empty, 996, 121–123, 521, 651–652, 661–662, 794 - Not working: 111–115, 124–126, 130–149, 151–153, 160, 163–169, 211–219, 221–222, 224–225, 231–232, 297–299, 412–414, 511, 522, 541, 700, 703, 704–719, 720–729, 730–739, 741–742, 750–752, 753–759, 760–762, 763–769, 771–774, 779, 781, 783–784, 791–792, 793–797, 810, 813–818, 870, 873–879, 881, 890–899 - Retired: 611, 621, 622, 786, 998, 785 |
| Income | Danish Income Statistics Registry | AEKVIVADISP_13 | We calculated annual household income after tax, adjusted for household size and inflation, using the median of the two years prior to breast cancer diagnosis. Quartiles were defined within five-year age groups, and income was categorized as low (Q1), intermediate (Q2–Q3), or high (Q4). If income data were missing for one of the two years, the available year was used; women with missing income in both years or with negative income in either year were excluded.^2^ |
| Stage at diagnosis | Danish Cancer Registry | TNM codes | As previously described.^3^ |
| Estrogen-receptor status | Danish Pathology Register | SNOMED codes | - ER+: F29521, or ÆKExxx (where xxx is % positivity). Before 2010: ER+ if ≥10%; after 2010: ER+ if ≥1% - ER–: F29522, or ÆKExxx (where xxx is % positivity). Before 2010: ER– if <10%; after 2010: ER– if <1% |
| Human epidermal growth factor receptor-2 (HER2) status | Danish Pathology Register | SNOMED codes | - HER2+: F29603 OR F29607 OR (F29602 HER2 borderline AND FE13B5 HER2 gene amplification) OR (F29606 AND FE13B5) - HER2−: (F29601 normal HER2) OR F29600 OR F29605 OR (F29606 AND FE13B1) OR (F29606 HER borderline AND FE13BF HER2 gene normal) |
| Charlson comorbidity index | National Patient Registry | ICD8 and ICD10 diagnosis codes | As previously described^4^ |
| Baseline psychiatric comorbidities | National Patient Registry and the Psychiatric Central Research Registry | ICD8 and ICD10 diagnosis codes | Categorization adapted from Møller *et al.*:^5^   - Severe psychiatric comorbidity: F20.0–20.9 OR F21.0–21.9 OR F22.0–29.9 OR F30.0–31.9 OR F60.31 - Moderate psychiatric comorbidity: F32.1 OR F32.2 OR F32.8–32.9 OR F33.1 OR F33.2 OR F33.4–33.9 OR F32.2 OR F32.3 OR F33.2 OR F33.3 OR F34.0–39.9 OR F40.0–41.9 OR F42.0–42.9 OR F43.0–43.9 OR F50.0–50.9 OR F60.0–60.30 OR F60.32–62.9 OR F90.0–90.9 OR F98.8C OR F91.0–92.9 |

# Table S2. Baseline sociodemographic and clinical characteristics^a^ of 74,711 opioid-naïve breast cancer survivors (study population three) in Denmark, 1997–2020.

| **Characteristics^a^** | **Opioid-naïve patients,**  **n (%)** |
| --- | --- |
| Total cohort | 74,711 (100) |
| *Sociodemographic characteristics* |  |
| Age at diagnosis (years) |  |
| <40 | 3,097 (4.1) |
| 40–49 | 10,424 (14) |
| 50–64 | 29,933 (40) |
| 65–74 | 18,364 (25) |
| ≥75 | 12,953 (17) |
| Marital status |  |
| Married | 44,040 (59) |
| Divorced | 10,466 (14) |
| Unmarried | 7,509 (10) |
| Widowed | 12,756 (17) |
| Cohabitation status |  |
| Cohabiting | 48,348 (65) |
| Living alone | 26,423 (35) |
| Education status |  |
| Short | 24,060 (32) |
| Intermediate | 26,421 (35) |
| Long | 20,256 (27) |
| Unknown | 4,034 (5.4) |
| Employment status |  |
| Working | 49,508 (66) |
| Not working | 10,334 (14) |
| Retired | 14,929 (20) |
| Household income, quartiles |  |
| Highest (>Q3) | 18,528 (25) |
| Intermediate (Q2–Q3) | 37,067 (50) |
| Lowest (<Q1) | 17,524 (25) |
| Unknown^b^ | 652 (0.9) |
|  |  |
| *Clinical characteristics* |  |
| Stage at diagnosis |  |
| Stage I | 39,222 (52) |
| Stage II | 26,871 (36) |
| Stage III | 8,678 (12) |
| Estrogen-receptor status (after 2005) ^c^ |  |
| Positive (ER+) | 33,969 (67) |
| Negative (ER-) | 10,994 (22) |
| Unknown/not measured | 5,888 (12) |
| HER2 status (after 2008) ^d^ |  |
| Positive | 5,037 (12) |
| Negative | 26,277 (63) |
| Unknown/not measured | 10,589 (25) |
| Charlson comorbidity index |  |
| None | 60,640 (81) |
| 1–2 | 12,309 (16) |
| ≥3 | 1,822 (2.4) |
| Psychiatric comorbidity |  |
| None | 72,324 (97) |
| Moderate | 1,650 (2.2) |
| Severe | 797 (1.1) |

Abbreviations: ER, estrogen receptor; HER2, human growth factor receptor 2

1. Characteristics were measured at or before the date of breast cancer diagnosis, except for ER and HER2 status, which were captured within 6 months after breast cancer diagnosis.
2. Women with missing or negative income data are categorized as unknown.
3. Mandatory reporting of pathological examinations to the Danish Pathology Register began in 2005. Counts in these cells will not sum to the total cohort size.
4. HER2 testing standardization began in 2008. Therefore, the numbers reported for HER2 testing are among the 41,903 patients diagnosed after the guideline change. Counts in these cells will not sum to the total cohort size


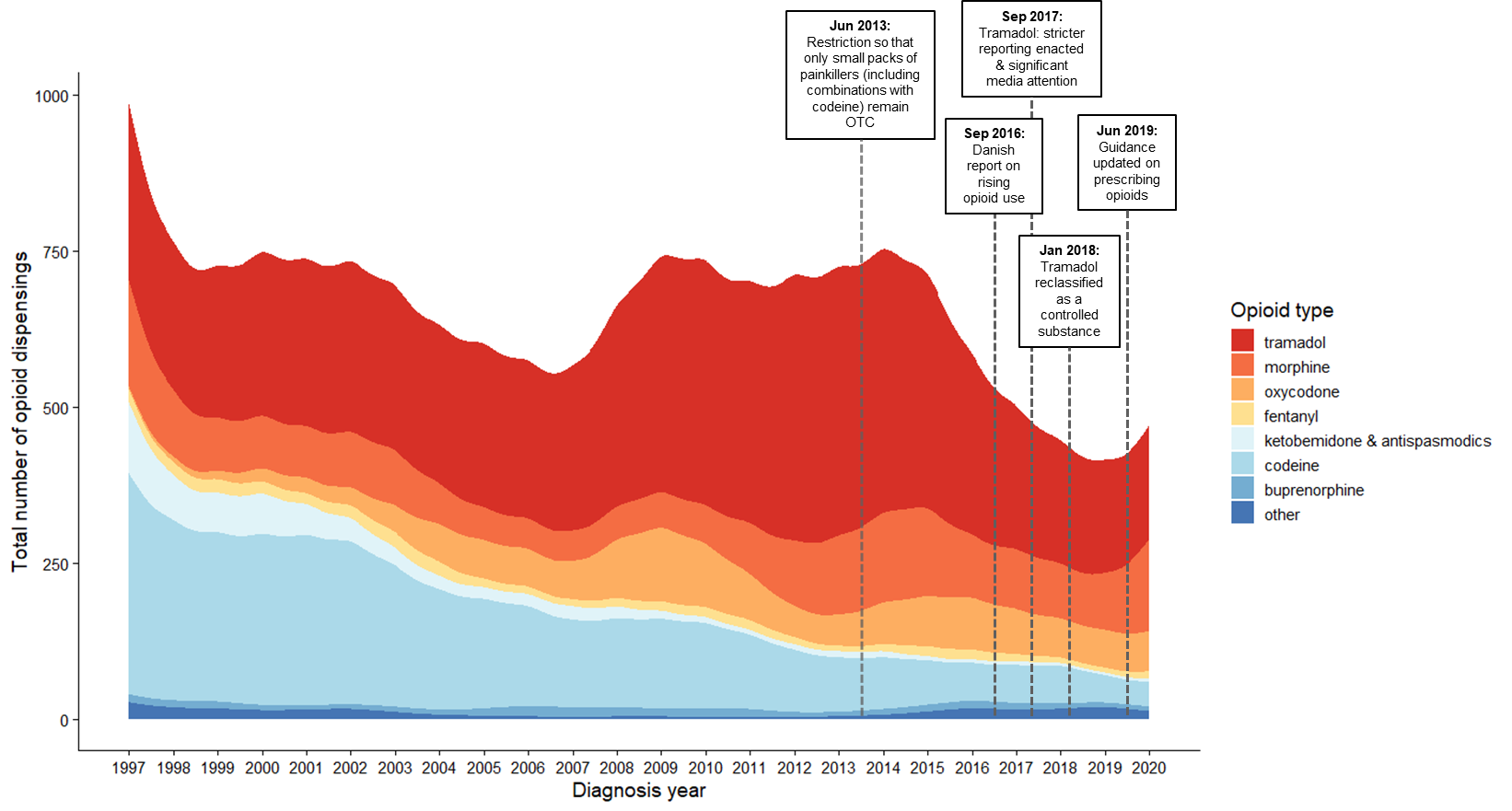


# Figure S1. Trends in all opioid prescribing within 6 months of breast cancer diagnosis among 74,711 opioid-naïve survivors (study population two) in Denmark, 1997–2020, shown in relation to national opioid policy and prescribing practice changes.

Abbreviations: OTC, over-the-counter

1. Each stream represents the total number of opioid prescriptions filled within 6 months of diagnosis, stratified by drug type.

**
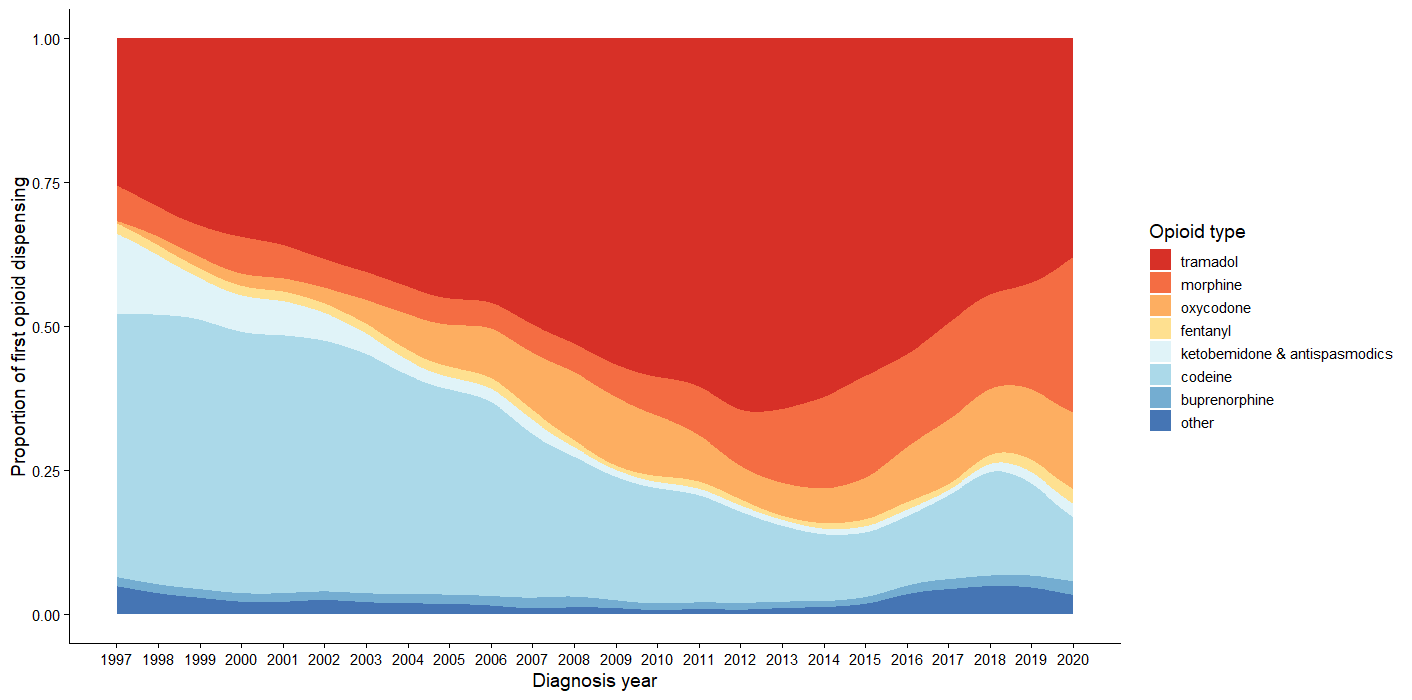
**

# Figure S2. Trends in initial opioid prescription within 6 months of breast cancer diagnosis among 74,711 opioid-naïve survivors (study population two) in Denmark, 1997–2020.

1. Each stream represents the proportion of survivors whose first opioid prescription within 6 months of diagnosis was for the specified drug. Proportions are based on only the first prescription and do not reflect subsequent changes in therapy.


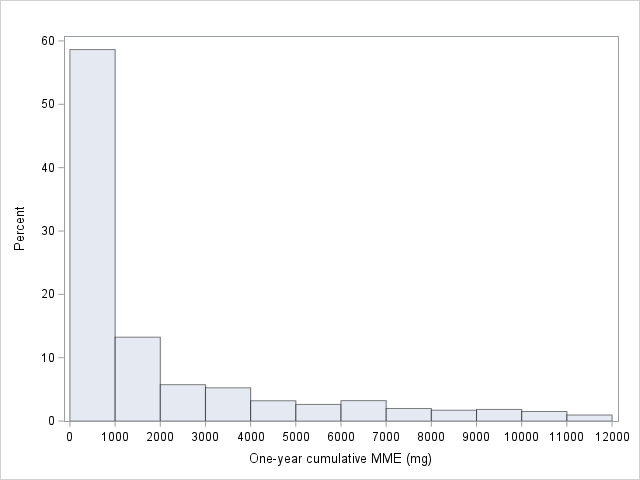


# Figure S3. Distribution of cumulative morphine milligram equivalents (MME) among 13,896 breast cancer survivors with at least one opioid dispensing in the year after diagnosis

1. Abbreviations: MME, morphine milligram equivalent
2. MME values are rounded to the nearest hundred.
3. The histogram is truncated at the 95^th^ percentile to improve visualization of the distribution.

# Table S3A. Associations of sociodemographic and clinical characteristics with four non-mutually exclusive opioid-related outcomes among 27,405 breast cancer survivors in Denmark, 1997–2004

|  | Long-term use | | Long-term high strength use | | Concurrent sedative-hypnotic use | | New and prolonged use^d^ | |
| --- | --- | --- | --- | --- | --- | --- | --- | --- |
| Characteristic | n (%) | OR (95% CI) | n (%) | OR (95% CI) | n (%) | OR (95% CI) | n (%) | OR (95% CI) |
| Total number of outcomes | 1,933 (7.5) | – | 683 (2.6) | – | 1,206 (4.7) | – | 529 (2.2) | – |
|  |  |  |  |  |  |  |  |  |
| Age at diagnosis^a^ |  |  |  |  |  |  |  |  |
| <40 | <35 | – | <20 | – | <20 | – | <15 | – |
| 40–49 | <150 | – | <55 | – | <120 | – | <60 | – |
| 50–64 | 649 (6.1) | Ref | 235 (2.2) | Ref | 427 (4.0) | Ref | 202 (2.1) | Ref |
| 65–74 | 542 (9.7) | 1.7 (1.5, 1.9) | 189 (3.4) | 1.5 (1.3, 1.9) | 334 (6.0) | 1.5 (1.3, 1.8) | 139 (2.8) | 1.3 (1.1, 1.7) |
| 75+ | 566 (12) | 2.1 (1.9, 2.4) | 196 (4.2) | 1.9 (1.6, 2.3) | 313 (6.7) | 1.7 (1.5, 2.0) | 122 (2.8) | 1.3 (1.1, 1.7) |
|  |  |  |  |  |  |  |  |  |
| Marital status^b^ |  |  |  |  |  |  |  |  |
| Married | 865 (5.7) | Ref | 297 (2.0) | Ref | 578 (3.8) | Ref | 256 (1.9) | Ref |
| Divorced | 347 (10) | 1.8 (1.6, 2.1) | 134 (3.9) | 2.0 (1.6, 2.4) | 241 (7.1) | 1.9 (1.6, 2.2) | 93 (3.1) | 1.6 (1.3, 2.1) |
| Unmarried | 134 (6.1) | 1.1 (0.9, 1.3) | 52 (2.4) | 1.2 (0.9, 1.7) | 57 (2.6) | 0.7 (0.5, 0.9) | 44 (2.1) | 1.1 (0.8, 1.6) |
| Widowed | 587 (11) | 1.2 (1.1, 1.4) | 200 (3.8) | 1.2 (1.0, 1.5) | 330 (6.3) | 1.2 (1.0, 1.4) | 136 (2.8) | 1.1 (0.9, 1.5) |
|  |  |  |  |  |  |  |  |  |
| Cohabitation^b^ |  |  |  |  |  |  |  |  |
| Cohabiting | 938 (5.8) | Ref | 326 (2.0) | Ref | 625 (3.8) | Ref | 292 (2.0) | Ref |
| Living alone | 995 (10) | 1.4 (1.3, 1.5) | 357 (3.7) | 1.4 (1.2, 1.7) | 581 (6.0) | 1.3 (1.1, 1.5) | 237 (2.7) | 1.2 (1.0, 1.4) |
|  |  |  |  |  |  |  |  |  |
| Education^b^ |  |  |  |  |  |  |  |  |
| Short | 942 (9.5) | 2.1 (1.8, 2.5) | 350 (3.6) | 2.3 (1.7, 2.9) | 529 (5.4) | 1.4 (1.2, 1.7) | 249 (2.8) | 1.8 (1.4, 2.4) |
| Medium | 476 (5.8) | 1.4 (1.2, 1.7) | 153 (1.9) | 1.3 (1.0, 1.8) | 335 (4.1) | 1.2 (1.0, 1.4) | 135 (1.8) | 1.3 (0.9, 1.7) |
| Long | 201 (3.8) | Ref | 69 (1.3) | Ref | 172 (3.3) | Ref | 69 (1.4) | Ref |
| Unknown | 314 (12) | 1.5 (1.2, 1.9) | 111 (4.2) | 1.7 (1.2, 2.4) | 170 (6.4) | 1.1 (0.8, 1.4) | 76 (2.8) | 1.4 (0.9, 2.1) |
|  |  |  |  |  |  |  |  |  |
| Employment^b^ |  |  |  |  |  |  |  |  |
| Working | 1,390 (6.8) | Ref | 507 (2.5) | Ref | 880 (4.3) | Ref | 408 (2.1) | Ref |
| Not working | 429 (12) | 2.7 (2.4, 3.0) | 144 (3.9) | 2.2 (1.8, 2.7) | 250 (6.8) | 2.1 (1.8, 2.4) | 89 (2.9) | 1.6 (1.3, 2.1) |
| Retired | 114 (6.8) | 0.9 (0.7, 1.1) | 32 (1.7) | 0.7 (0.5, 1.0) | 76 (4.1) | 0.9 (0.7, 1.2) | 32 (1.9) | 0.9 (0.6, 1.2) |
|  |  |  |  |  |  |  |  |  |
| Household income^a^ |  |  |  |  |  |  |  |  |
| Highest (>Q3) | <180 | – | <65 | – | <145 | – | <70 | – |
| Middle (Q1–Q3) | 699 (5.8) | Ref | 235 (1.9) | Ref | 481 (4.0) | Ref | 190 (1.7) | Ref |
| Lowest (<Q1) | 1,058 (11) | 2.0 (1.8, 2.2) | 387 (4.0) | 2.1 (1.8, 2.5) | 578 (6.0) | 1.5 (1.4, 1.8) | 274 (3.1) | 1.9 (1.6, 2.3) |
| Unknown | <10 | – | <5 | – | <10 | – | <5 | – |
|  |  |  |  |  |  |  |  |  |
| Stage at diagnosis^b^ |  |  |  |  |  |  |  |  |
| Stage I | 863 (6.5) | Ref | 241 (1.8) | Ref | 569 (4.3) | Ref | 206 (1.7) | Ref |
| Stage II | 806 (7.2) | 1.2 (1.1, 1.3) | 272 (2.4) | 1.4 (1.2, 1.7) | 515 (4.6) | 1.1 (1.0, 1.3) | 210 (2.0) | 1.2 (1.0, 1.5) |
| Stage III | 264 (19) | 3.4 (2.9, 3.9) | 170 (12) | 7.5 (6.1, 9.2) | 122 (8.9) | 2.1 (1.7, 2.6) | 113 (6.3) | 3.6 (2.8, 4.5) |
|  |  |  |  |  |  |  |  |  |
| Estrogen receptor^c^ |  |  |  |  |  |  |  |  |
| Positive | – | – | – | – | – | – | – | – |
| Negative | – | – | – | – | – | – | – | – |
| Unknown | – | – | – | – | – | – | – | – |
|  |  |  |  |  |  |  |  |  |
| HER2 status^c^ |  |  |  |  |  |  |  |  |
| Positive | – | – | – | – | – | – | – | – |
| Negative | – | – | – | – | – | – | – | – |
| Unknown | – | – | – | – | – | – | – | – |
|  |  |  |  |  |  |  |  |  |
| Charlson comorbidity index^b^ |  |  |  |  |  |  |  |  |
| None | 1,306 (6.0) | Ref | 451 (2.1) | Ref | 872 (4.0) | Ref | – | Ref |
| 1 or 2 | 541 (14) | 2.2 (2.0, 2.4) | 195 (5.1) | 2.2 (1.8, 2.6) | 287 (7.5) | 1.7 (1.5, 2.0) | – | – |
| 3 or more | 86 (20) | 3.0 (2.4, 3.9) | 37 (8.6) | 3.5 (2.5, 5.0) | 47 (11) | 2.4 (1.8, 3.3) | – | – |
|  |  |  |  |  |  |  |  |  |

1. Reported associations are unadjusted.
2. Reported associations are adjusted for age at diagnosis.
3. Mandatory of reporting of pathological examinations to the Danish Pathology Register began in 2005 and HER2 testing in 2008.
4. New and prolonged use was investigated among 23,790 opioid-naive patients.
5. Cells with small counts and any cells that allow for back-calculation are masked in accordance with the General Data Protection Regulation.

# Table S3B. Associations of sociodemographic and clinical characteristics with four non-mutually exclusive opioid-related outcomes among 31,266 breast cancer survivors in Denmark, 2005–2012

|  | Long-term use | | Long-term high strength use | | Concurrent sedative-hypnotic use | | New and prolonged use | |
| --- | --- | --- | --- | --- | --- | --- | --- | --- |
| Characteristic | n (%) | OR (95% CI) | n (%) | OR (95% CI) | n (%) | OR (95% CI) | n (%) | OR (95% CI) |
| Total number of outcomes | 1,993 (6.4) | – | 605 (1.9) | – | 1,141 (3.6) | – | 428 (1.6) | – |
|  |  |  |  |  |  |  |  |  |
| Age at diagnosis^a^ |  |  |  |  |  |  |  |  |
| <40 | <30 | – | <10 | – | <20 | – | <10 | – |
| 40–49 | <150 | – | <50 | – | <105 | – | <55 | – |
| 50–64 | 685 (5.3) | Ref | 190 (1.5) | Ref | 401 (3.1) | Ref | 163 (1.4) | Ref |
| 65–74 | 554 (6.8) | 1.3 (1.2, 1.5) | 171 (2.1) | 1.5 (1.2, 1.8) | 347 (4.3) | 1.4 (1.2, 1.6) | 110 (1.6) | 1.1 (0.9, 1.4) |
| 75+ | 586 (12) | 2.4 (2.2, 2.7) | 195 (4.0) | 2.8 (2.3, 3.4) | 278 (5.7) | 1.9 (1.6, 2.2) | 99 (2.5) | 1.8 (1.4, 2.3) |
|  |  |  |  |  |  |  |  |  |
| Marital status^b^ |  |  |  |  |  |  |  |  |
| Married | 918 (5.0) | Ref | 263 (1.4) | Ref | 516 (2.8) | Ref | 221 (1.4) | Ref |
| Divorced | 387 (8.6) | 1.8 (1.6, 2.0) | 125 (2.8) | 1.9 (1.5, 2.4) | 233 (5.2) | 1.9 (1.6, 2.2) | 83 (2.2) | 1.6 (1.3, 2.1) |
| Unmarried | 158 (5.4) | 1.3 (1.1, 1.6) | 43 (1.5) | 1.3 (0.9, 1.7) | 108 (3.7) | 1.5 (1.2, 1.9) | 40 (1.5) | 1.3 (0.9, 1.8) |
| Widowed | 530 (10) | 1.3 (1.2, 1.5) | 174 (3.3) | 1.4 (1.1, 1.7) | 284 (5.4) | 1.4 (1.2, 1.7) | 84 (1.9) | 1.1 (0.8, 1.4) |
|  |  |  |  |  |  |  |  |  |
| Cohabitation^b^ |  |  |  |  |  |  |  |  |
| Cohabiting | 1,020 (5.0) | Ref | 291 (1.4) | Ref | 562 (2.8) | Ref | 239 (1.3) | Ref |
| Living alone | 973 (8.9) | 1.4 (1.3, 1.6) | 314 (2.9) | 1.5 (1.3, 1.8) | 579 (5.3) | 1.7 (1.5, 1.9) | 189 (2.1) | 1.4 (1.1, 1.7) |
|  |  |  |  |  |  |  |  |  |
| Education^b^ |  |  |  |  |  |  |  |  |
| Short | 992 (9.3) | 2.1 (1.8, 2.4) | 310 (2.9) | 2.1 (1.7, 2.7) | 502 (4.7) | 1.5 (1.2, 1.7) | 183 (2.1) | 1.6 (1.2, 2.1) |
| Medium | 586 (5.2) | 1.3 (1.1, 1.5) | 171 (1.5) | 1.3 (1.0, 1.7) | 356 (3.1) | 1.1 (0.9, 1.3) | 134 (1.4) | 1.1 (0.8, 1.5) |
| Long | 307 (3.8) | Ref | 85 (1.0) | Ref | 221 (2.7) | Ref | 87 (1.2) | Ref |
| Unknown | 108 (11) | 1.8 (1.4, 2.3) | 39 (3.9) | 2.0 (1.3, 3.1) | 62 (6.1) | 1.6 (1.2, 2.2) | 24 (2.7) | 1.8 (1.1, 2.9) |
|  |  |  |  |  |  |  |  |  |
| Employment^b^ |  |  |  |  |  |  |  |  |
| Working | 1,058 (5.0) | Ref | 325 (1.5) | Ref | 614 (2.9) | Ref | 269 (1.4) | Ref |
| Not working | 670 (12) | 3.8 (3.4, 4.3) | 217 (4.0) | 4.3 (3.5, 5.2) | 373 (6.9) | 3.2 (2.7, 3.6) | 102 (2.5) | 2.0 (1.6, 2.5) |
| Retired | 265 (5.7) | 0.9 (0.8, 1.0) | 63 (1.4) | 0.7 (0.5, 0.9) | 154 (3.3) | 0.9 (0.8, 1.1) | 57 (1.4) | 0.8 (0.6, 1.1) |
|  |  |  |  |  |  |  |  |  |
| Household income^a^ |  |  |  |  |  |  |  |  |
| Highest (>Q3) | <290 | – | <90 | – | <195 | – | <70 | – |
| Middle (Q1–Q3) | 1,020 (6.3) | Ref | 310 (1.9) | Ref | 584 (3.6) | Ref | 207 (1.5) | Ref |
| Lowest (<Q1) | 681 (9.3) | 1.5 (1.4, 1.7) | 213 (2.9) | 1.5 (1.3, 1.8) | 362 (4.9) | 1.4 (1.2, 1.6) | 156 (2.5) | 1.7 (1.4, 2.1) |
| Unknown | <10 | – | <5 | – | <5 | – | <5 | – |
|  |  |  |  |  |  |  |  |  |
| Stage at diagnosis^b^ |  |  |  |  |  |  |  |  |
| Stage I | 851 (5.4) | Ref | 229 (1.5) | Ref | 494 (3.1) | Ref | 166 (1.2) | Ref |
| Stage II | 722 (6.7) | 1.2 (1.1, 1.3) | 211 (2.0) | 1.2 (1.0, 1.5) | 416 (3.9) | 1.2 (1.0, 1.3) | 157 (1.7) | 1.3 (1.1, 1.7) |
| Stage III | 420 (8.9) | 1.6 (1.4, 1.8) | 165 (3.5) | 2.2 (1.8, 2.7) | 231 (4.9) | 1.5 (1.3, 1.8) | 105 (2.5) | 2.0 (1.6, 2.6) |
|  |  |  |  |  |  |  |  |  |
| Estrogen receptor^b,c^ |  |  |  |  |  |  |  |  |
| Positive | 1,204 (6.5) | Ref | 360 (1.9) | Ref | 705 (3.8) | Ref | 253 (1.6) | Ref |
| Negative | 594 (6.3) | 1.0 (0.9, 1.2) | 175 (1.9) | 1.0 (0.9, 1.3) | 323 (3.4) | 1.0 (0.8, 1.1) | 135 (1.7) | 1.1 (0.9, 1.3) |
| Unknown | 195 (6.0) | 0.9 (0.8, 1.1) | 70 (2.1) | 1.1 (0.9, 1.5) | 113 (3.5) | 0.9 (0.8, 1.1) | 40 (1.4) | 0.9 (0.6, 1.2) |
|  |  |  |  |  |  |  |  |  |
| HER2 status^b,c^ |  |  |  |  |  |  |  |  |
| Positive | 157 (5.8) | 1.0 (0.9, 1.2) | 47 (1.7) | 1.2 (0.9, 1.7) | 99 (3.7) | 1.2 (1.0, 1.5) | 52 (1.5) | 1.0 (0.7, 1.3) |
| Negative | 794 (6.1) | Ref | 203 (1.6) | Ref | 422 (3.2) | Ref | 241 (1.6) | Ref |
| Unknown | 325 (6.2) | 1.0 (0.8, 1.1) | 114 (2.2) | 1.3 (1.0, 1.6) | 190 (3.6) | 1.1 (0.9, 1.3) | 135 (1.6) | 1.0 (0.8, 1.2) |
|  |  |  |  |  |  |  |  |  |
| Charlson comorbidity index^b^ |  |  |  |  |  |  |  |  |
| None | 1,332 (5.0) | Ref | 372 (1.4) | Ref | 822 (3.1) | Ref | – | Ref |
| 1 or 2 | 550 (14) | 2.5 (2.2, 2.8) | 186 (4.6) | 2.8 (2.3, 3.3) | 262 (6.5) | 1.9 (1.6, 2.2) | – | – |
| 3 or more | 111 (23) | 4.3 (3.4, 5.4) | 47 (9.5) | 5.6 (4.0, 7.7) | 57 (12) | 3.4 (2.5, 4.5) | – | – |
|  |  |  |  |  |  |  |  |  |

1. Reported associations are unadjusted.
2. Reported associations are adjusted for age at diagnosis.
3. Mandatory of reporting of pathological examinations to the Danish Pathology Register began in 2005 and HER2 testing in 2008.
4. New and prolonged use was investigated among 27,061 opioid-naive patients.
5. Cells with small counts and any cells that allow for back-calculation are masked in accordance with the General Data Protection Regulation.

# Table S3C. Associations of sociodemographic and clinical characteristics with four non-mutually exclusive opioid-related outcomes among 27,405 breast cancer survivors in Denmark, 2013–2020

|  | Long-term use | | Long-term high strength use | | Concurrent sedative-hypnotic use | | New and prolonged use | |
| --- | --- | --- | --- | --- | --- | --- | --- | --- |
| Characteristic | n (%) | OR (95% CI) | n (%) | OR (95% CI) | n (%) | OR (95% CI) | n (%) | OR (95% CI) |
| Total number of outcomes | 1,269 (4.6) | – | 392 (1.4) | – | 684 (2.5) | – | 322 (1.2) | – |
|  |  |  |  |  |  |  |  |  |
| Age at diagnosis |  |  |  |  |  |  |  |  |
| <40 | <30 | – | <10 | – | <20 | – | <10 | – |
| 40–49 | <105 | – | <35 | – | <70 | – | <45 | – |
| 50–64 | 322 (3.2) | Ref | 92 (0.9) | Ref | 191 (1.9) | Ref | 94 (1.1) | Ref |
| 65–74 | 394 (5.2) | 1.6 (1.4, 1.9) | 110 (1.5) | 1.6 (1.2, 2.1) | 218 (2.9) | 1.5 (1.2, 1.8) | 81 (1.3) | 1.2 (0.9, 1.6) |
| 75+ | 433 (7.8) | 2.5 (2.2, 2.9) | 159 (2.9) | 3.2 (2.4, 4.1) | 196 (3.5) | 1.9 (1.5, 2.3) | 101 (2.2) | 2.1 (1.6, 2.8) |
|  |  |  |  |  |  |  |  |  |
| Marital status |  |  |  |  |  |  |  |  |
| Married | 564 (3.6) | Ref | 173 (1.1) | Ref | 310 (2.0) | Ref | 158 (1.1) | Ref |
| Divorced | 264 (6.0) | 1.7 (1.5, 2.0) | 80 (1.8) | 1.6 (1.3, 2.1) | 155 (3.5) | 1.8 (1.5, 2.2) | 63 (1.7) | 1.5 (1.1, 2.0) |
| Unmarried | 114 (3.7) | 1.3 (1.0, 1.6) | 29 (0.9) | 1.1 (0.7, 1.6) | 69 (2.2) | 1.3 (1.0, 1.7) | 36 (1.3) | 1.3 (0.9, 1.9) |
| Widowed | 327 (7.7) | 1.5 (1.3, 1.7) | 110 (2.6) | 1.4 (1.1, 1.8) | 150 (3.6) | 1.4 (1.1, 1.7) | 65 (1.9) | 1.2 (0.9, 1.6) |
|  |  |  |  |  |  |  |  |  |
| Cohabitation |  |  |  |  |  |  |  |  |
| Cohabiting | 628 (3.6) | Ref | 193 (1.1) | Ref | 346 (2.0) | Ref | 182 (1.2) | Ref |
| Living alone | 641 (6.5) | 1.5 (1.4, 1.7) | 199 (2.0) | 1.4 (1.1, 1.7) | 338 (3.4) | 1.6 (1.3, 1.8) | 140 (1.7) | 1.3 (1.0, 1.6) |
|  |  |  |  |  |  |  |  |  |
| Education |  |  |  |  |  |  |  |  |
| Short | 567 (7.3) | 2.2 (1.8, 2.5) | 194 (2.5) | 2.3 (1.7, 3.1) | 270 (3.5) | 1.5 (1.2, 1.8) | 115 (1.8) | 1.5 (1.1, 2.0) |
| Medium | 428 (4.1) | 1.4 (1.2, 1.6) | 121 (1.2) | 1.3 (1.0, 1.7) | 222 (2.1) | 1.0 (0.8, 1.2) | 117 (1.3) | 1.2 (0.9, 1.6) |
| Long | 243 (2.8) | Ref | 71 (0.8) | Ref | 178 (2.0) | Ref | 82 (1.0) | Ref |
| Unknown | 31 (6.0) | 1.9 (1.3, 2.8) | 6 (1.2) | 1.2 (0.5, 2.7) | 14 (2.7) | 1.2 (0.7, 2.1) | 8 (1.7) | 1.5 (0.7, 3.1) |
|  |  |  |  |  |  |  |  |  |
| Employment |  |  |  |  |  |  |  |  |
| Working | 217 (1.8) | Ref | 46 (0.4) | Ref | 159 (1.3) | Ref | 95 (0.8) | Ref |
| Not working | 360 (8.9) | 5.1 (4.3, 6.1) | 118 (2.9) | 7.4 (5.2, 10) | 184 (4.6) | 3.5 (2.8, 4.3) | 58 (1.9) | 2.2 (1.6, 3.1) |
| Retired | 692 (6.3) | 1.9 (1.5, 2.3) | 228 (2.1) | 2.3 (1.5, 3.5) | 341 (3.1) | 1.5 (1.2, 2.0) | 169 (1.8) | 1.8 (1.2, 2.6) |
|  |  |  |  |  |  |  |  |  |
| Household income |  |  |  |  |  |  |  |  |
| Highest (>Q3) | <265 | – | <80 | – | <180 | – | <85 | – |
| Middle (Q1–Q3) | 779 (5.7) | Ref | 254 (1.9) | Ref | 398 (2.9) | Ref | 193 (1.6) | Ref |
| Lowest (<Q1) | 224 (5.5) | 1.0 (0.8, 1.1) | 67 (1.7) | 0.9 (0.7, 1.2) | 109 (2.7) | 0.9 (0.7, 1.2) | 47 (1.3) | 0.8 (0.6, 1.1) |
| Unknown | <10 | – | <5 | – | <10 | – | <5 | – |
|  |  |  |  |  |  |  |  |  |
| Stage at diagnosis |  |  |  |  |  |  |  |  |
| Stage I | 679 (4.2) | Ref | 200 (1.2) | Ref | 359 (2.2) | Ref | 147 (1.1) | Ref |
| Stage II | 397 (4.8) | 1.1 (0.9, 1.2) | 115 (1.4) | 1.0 (0.8, 1.3) | 236 (2.9) | 1.2 (1.0, 1.5) | 109 (1.5) | 1.4 (1.1, 1.8) |
| Stage III | 193 (6.4) | 1.4 (1.2, 1.7) | 77 (2.6) | 1.8 (1.4, 2.4) | 89 (3.0) | 1.3 (1.0, 1.6) | 66 (2.5) | 2.2 (1.6, 3.0) |
|  |  |  |  |  |  |  |  |  |
| Estrogen receptor |  |  |  |  |  |  |  |  |
| Positive | 933 (4.5) | Ref | 267 (1.3) | Ref | 497 (2.4) | Ref | 232 (1.3) | Ref |
| Negative | 155 (5.0) | 1.2 (1.0, 1.4) | 58 (1.9) | 1.6 (1.2, 2.1) | 108 (3.5) | 1.5 (1.2, 1.9) | 47 (1.7) | 1.4 (1.0, 1.9) |
| Unknown | 181 (5.2) | 1.1 (0.9, 1.3) | 67 (1.9) | 1.4 (1.1, 1.8) | 79 (2.3) | 0.9 (0.7, 1.2) | 43 (1.4) | 1.1 (0.8, 1.5) |
|  |  |  |  |  |  |  |  |  |
| HER2 status |  |  |  |  |  |  |  |  |
| Positive | 123 (4.0) | 1.0 (0.8, 1.2) | 43 (1.4) | 1.1 (0.8, 1.6) | 79 (2.6) | 1.1 (0.9, 1.4) | 39 (1.4) | 1.2 (0.8, 1.6) |
| Negative | 779 (4.5) | Ref | 235 (1.4) | Ref | 433 (2.5) | Ref | 196 (1.3) | Ref |
| Unknown | 367 (5.2) | 1.1 (1.0, 1.3) | 114 (1.6) | 1.2 (0.9, 1.5) | 172 (2.5) | 1.0 (0.8, 1.2) | 87 (1.4) | 1.1 (0.8, 1.4) |
|  |  |  |  |  |  |  |  |  |
| Charlson comorbidity index |  |  |  |  |  |  |  |  |
| None | 867 (3.8) | Ref | 244 (1.1) | Ref | 494 (2.2) | Ref | – | Ref |
| 1 or 2 | 340 (8.4) | 1.9 (1.7, 2.2) | 122 (3.0) | 2.3 (1.9, 2.9) | 163 (4.0) | 1.7 (1.4, 2.0) | – | – |
| 3 or more | 62 (11) | 2.4 (1.8, 3.2) | 26 (4.6) | 3.3 (2.1, 5.0) | 27 (4.7) | 1.9 (1.3, 2.8) | – | – |
|  |  |  |  |  |  |  |  |  |

1. Reported associations are unadjusted.
2. Reported associations are adjusted for age at diagnosis.
3. New and prolonged use was investigated among 23,790 opioid-naive patients.
4. Cells with small counts and any cells that allow for back-calculation are masked in accordance with the General Data Protection Regulation.

# References

1. Hjorth CF, Kjærulff TM, Thomsen MK, et al. SEPLINE: Socioeconomic Position in Epidemiological Research-A National Guideline on Danish Registry Data. *Clin Epidemiol*. 2025;17:593-624. doi:10.2147/CLEP.S520772

2. Hjorth CF, Damkier P, Ejlertsen B, Lash T, Sørensen HT, Cronin-Fenton D. Socioeconomic position and prognosis in premenopausal breast cancer: a population-based cohort study in Denmark. *BMC Med*. 2021;19:235. doi:10.1186/s12916-021-02108-z

3. Ording AG, Nielsson MS, Frøslev T, Friis S, Garne JP, Søgaard M. Completeness of breast cancer staging in the Danish Cancer Registry, 2004–2009. *Clin Epidemiol*. 2012;4(Suppl 2):11-16. doi:10.2147/CLEP.S31574

4. Pedersen MK, Eriksson R, Reguant R, et al. A unidirectional mapping of ICD-8 to ICD-10 codes, for harmonized longitudinal analysis of diseases. *Eur J Epidemiol*. 2023;38(10):1043-1052. doi:10.1007/s10654-023-01027-y

5. Møller JJK, la Cour K, Pilegaard MS, Möller S, Jarlbaek L. Identification of socially vulnerable cancer patients - development of a register-based index (rSVI). *Support Care Cancer*. 2022;30(6):5277-5287. doi:10.1007/s00520-022-06937-3
